# Internet and Computer-Based Cognitive Behavioral Therapy for Anxiety and Depression in Adolescents and Young Adults: Systematic Review and Meta-Analysis

**DOI:** 10.2196/17831

**Published:** 2020-09-25

**Authors:** Carolien Christ, Maria JE Schouten, Matthijs Blankers, Digna JF van Schaik, Aartjan TF Beekman, Marike A Wisman, Yvonne AJ Stikkelbroek, Jack JM Dekker

**Affiliations:** 1 Department of Psychiatry GGZ inGeest Amsterdam UMC, Vrije Universiteit Amsterdam Amsterdam Netherlands; 2 Department of Research Arkin Mental Health Care Amsterdam Netherlands; 3 Amsterdam Public Health Research Institute Amsterdam UMC Vrije Universiteit Amsterdam Amsterdam Netherlands; 4 Trimbos institute-The Netherlands Institute of Mental Health and Addiction Utrecht Netherlands; 5 Department of Youth and Family Arkin Mental Health Care Amsterdam Netherlands; 6 Department of Child and Adolescent Studies Universiteit Utrecht Utrecht Netherlands; 7 Depression Expert Center for Youth Mental Health Care Oost-Brabant Boekel Netherlands; 8 Department of Clinical Psychology Vrije Universiteit Amsterdam Amsterdam Netherlands

**Keywords:** cognitive behavior therapy, internet, anxiety, depression, youth, meta-analysis

## Abstract

**Background:**

Anxiety and depressive disorders are prevalent in adolescents and young adults. However, most young people with mental health problems do not receive treatment. Computerized cognitive behavior therapy (cCBT) may provide an accessible alternative to face-to-face treatment, but the evidence base in young people is limited.

**Objective:**

The objective was to perform an up-to-date comprehensive systematic review and meta-analysis of the effectiveness of cCBT in treating anxiety and depression in adolescents and young adults compared with active treatment and passive controls. We aimed to examine posttreatment and follow-up effects and explore the moderators of treatment effects.

**Methods:**

We conducted systematic searches in the following six electronic databases: PubMed, EMBASE, PsycINFO, CINAHL, Web of Science, and Cochrane Central Register of Controlled Trials. We included randomized controlled trials comparing cCBT with any control group in adolescents or young adults (age 12-25 years) with anxiety or depressive symptoms. The quality of included studies was assessed using the Cochrane risk-of-bias tool for randomized trials, version 2.0. Overall quality of evidence for each outcome was assessed using the Grading of Recommendations Assessment, Development and Evaluation (GRADE) approach. Posttreatment means and SDs were compared between intervention and control groups, and pooled effect sizes (Hedges *g*) were calculated. Random-effects meta-analyses were conducted using Comprehensive Meta-Analysis software. Subgroup analyses and meta-regression analyses were conducted to explore whether age, guidance level, and adherence rate were associated with treatment outcome.

**Results:**

The search identified 7670 papers, of which 24 studies met the inclusion criteria. Most included studies (22/24) had a high risk of bias owing to self-report measures and/or inappropriate handling of missing data. Compared with passive controls, cCBT yielded small to medium posttreatment pooled effect sizes regarding depressive symptoms (*g*=0.51, 95% CI 0.30-0.72, number needed to treat [NNT]=3.55) and anxiety symptoms (*g*=0.44, 95% CI 0.23-0.65, NNT=4.10). cCBT yielded effects similar to those of active treatment controls regarding anxiety symptoms (*g*=0.04, 95% CI −0.23 to 0.31). For depressive symptoms, the nonsignificant pooled effect size favored active treatment controls (*g*=−0.70, 95% CI −1.51 to 0.11, *P*=.09), but heterogeneity was very high (*I^2^*=90.63%). No moderators of treatment effects were identified. At long-term follow-up, cCBT yielded a small pooled effect size regarding depressive symptoms compared with passive controls (*g*=0.27, 95% CI 0.09-0.45, NNT=6.58). No other follow-up effects were found; however, power was limited owing to the small number of studies.

**Conclusions:**

cCBT is beneficial for reducing posttreatment anxiety and depressive symptoms in adolescents and young adults compared with passive controls. Compared with active treatment controls, cCBT yielded similar effects regarding anxiety symptoms. Regarding depressive symptoms, however, the results remain unclear. More high-quality research involving active controls and long-term follow-up assessments is needed in this population.

**Trial Registration:**

PROSPERO CRD42019119725; https://tinyurl.com/y5acfgd9.

## Introduction

Anxiety and depressive disorders are common in children and adolescents [1,2]. Symptoms of anxiety and depression in childhood and adolescence predict a range of mental health problems later in life, including adult anxiety and depressive disorders and substance use disorders [3-8]. Moreover, anxiety and depressive disorders in young people are associated with an increased risk of self-harm and suicide [5,9], the second most common cause of death among youth aged between 10 and 24 years [10].

Among children and adolescents aged up to 18 years, global prevalence rates are estimated at 6.5% for anxiety disorders and 2.6% for depressive disorders [11]. The prevalence of mental disorders increases during the transition from childhood to adolescence [12,13], with prevalence rates in adolescents (ie, age 12-19 years) estimated at 10.7% for anxiety disorders and 6.1% for depressive disorders [13]. The incidence of most anxiety disorders peaks during adolescence, whereas the incidence of depressive disorders starts to rise during adolescence [14] and peaks in young adulthood (ie, age 19-25 years) [15-17]. Given the high incidence and burden of anxiety and depressive disorders in young people, early intervention in both adolescents and young adults is of utmost importance.

Adolescents and young adults with anxiety or depressive disorders are commonly treated with cognitive behavioral therapy (CBT), which is a widely-used treatment that has been proven to be effective in this population [18-21]. However, the majority of adolescents and young adults with mental health problems do not receive treatment [22-25]. Among their reasons for low treatment utilization are limited availability of youth mental health services, perceived stigma associated with mental illness, perceived lack of time or resources, and preference for self-help [24,26,27]. These barriers to treatment utilization may partly be overcome by computerized mental health interventions involving psychological treatment delivered via the internet and/or digital devices. Compared with face-to-face treatment, computerized interventions may provide more flexible access in terms of time, location, and availability; greater privacy and anonymity; and more independence [28,29]. The internet is ubiquitous in the lives of young people, who have shown positive attitudes toward computerized mental health interventions [30]. Therefore, computerized treatment provides an accessible and feasible alternative to face-to-face treatment for this group [31,32].

Numerous randomized controlled trials (RCTs) and meta-analyses in adult populations with anxiety and depressive disorders have shown that CBT may be effectively delivered via the internet or digital devices [33,34]. The effects of these so-called computerized CBT (cCBT) interventions have been demonstrated to be comparable to the effects of face-to-face CBT in adults [35]. In children and young people, cCBT has been found to be effective in treating mental health problems as well [36-42]. Despite these promising results, however, the evidence base on cCBT in young people remains limited compared with research in adults. The number of studies is still small, and the quality of RCTs is often low [39].

To date, three meta-analyses have shown cCBT [36,37] and internet-based mental health interventions [43] to be effective in reducing anxiety and depressive symptoms in young people aged 12 to 25 years. Ebert et al [36] found cCBT to be superior to passive control conditions for both anxiety (*g*=0.68, 95% CI 0.45-0.92, *P*<.001; *k*=7) and depression (*g*=0.76, 95% CI 0.23-2.66, *P*<.001; *k*=4). Active control conditions were not included in their meta-analysis. Similarly, Pennant et al [37] found positive effects of cCBT on both anxiety (standardized mean difference [SMD]=−0.77, 95% CI −1.45 to −0.09, *k*=6) and depression (SMD=−0.62, 95% CI −1.13 to −0.11, *k*=7) compared with passive controls. Compared with face-to-face CBT, their meta-analysis showed similar effects for cCBT on anxiety (SMD=−0.04, 95% CI −0.36 to 0.28, *P=*.89; *k*=3), but a large effect in favor of face-to-face CBT on depression (SMD=1.65, 95% CI 0.88-2.41, *P*<.001; *k*=2). However, these meta-analyses included only a small number of studies that were all published up to 2013. A more recent meta-analysis in children and adolescents up to 18 years with depressive and/or anxiety symptoms showed that cCBT interventions yielded a medium effect size compared with waiting list controls (*g*=0.66, 95% CI 0.42-0.90, *P*<.001, *k*=17) [44]. This study reported neither separate effects of cCBT on depression and anxiety symptoms nor effects of cCBT compared with face-to-face CBT. Importantly, none of these meta-analyses reported mid-term or long-term effects [36,37,44].

To our knowledge, the study of Välimäki et al [43] is the only meta-analysis that not only reported posttreatment effects, but also described short-term and long-term follow-up effects. Posttreatment effects showed the positive effects of internet-based interventions on depressive symptoms (*P*=.02, median=1.68, 95% CI 0.25-3.11, *k*=10) and anxiety symptoms (*P*=.001, median=1.47, 95% CI 0.59-2.36, *k*=8) compared with any control group. The authors found significant long-term effects of internet-based interventions aimed at reducing depressive symptoms 6 months after treatment (*P*=.01, median=1.78, 95% CI 0.37-3.20, *k=*3), but no mid-term effects (ie, 3-5 months after treatment). Regarding anxiety symptoms, they found no mid-term effects in the only two available studies, and no study reported long-term results on anxiety [43]. However, their meta-analysis included both cCBT and various other internet-based mental health interventions (eg, positive psychology), and did not specifically analyze the effects of cCBT. Furthermore, the effects of internet-based interventions were not reported separately compared with active treatment controls and passive controls. In addition, the authors used a narrow search string, which did not include anxiety disorders or interventions aimed at decreasing anxiety symptoms. Hence, it remains unclear whether cCBT is effective in treating young people with anxiety and depressive disorders in the long term, compared with active treatment and passive controls.

In adults, individual participant data meta-analyses on internet-based interventions have demonstrated several predictors of better treatment outcomes, among which are older age [45] and higher treatment adherence [33]. In addition, level of guidance (ie, the level of therapist support provided during cCBT) appears to be associated with larger treatment effects in adults, as studies on guided internet-based interventions have generally demonstrated larger effect sizes than studies on unguided interventions [33,45,46]. Although previous meta-analyses on cCBT in children and young people have attempted to identify moderators of treatment effects, the results remain mixed. Some found evidence for a moderating role of age [36,37,42], whereas others did not [38,44]. With regard to guidance, evidence remains mixed as well [37,42,44]. To our knowledge, previous meta-analyses in young people did not examine whether treatment adherence is associated with cCBT effect sizes.

Research on cCBT in young people with anxiety or depressive symptoms is a rapidly developing field, and all previous meta-analyses are limited to studies of at least 2 years old [36-38,43,44]. In addition, the most recent meta-analyses focused on other age groups [38,41,42,44] or did not separately report effects for either anxiety and depressive disorders [38,44] or cCBT [43]. Moreover, the follow-up effects of cCBT remain largely unknown. Lastly, since most previous meta-analyses in young people did not separately compare cCBT to active treatment and passive controls [36,43], it remains unclear whether cCBT provides an effective alternative to face-to-face treatment in this group. Therefore, our objective was to provide an up-to-date comprehensive systematic review and meta-analysis of the effectiveness of cCBT in treating anxiety and depressive symptoms in adolescents and young adults compared with active treatment and passive control groups, differentiating between posttreatment, short-term follow-up, and long-term follow-up. Furthermore, we aimed to explore whether age, guidance level, and treatment adherence are associated with treatment outcome by conducting subgroup analyses and meta-regression analyses.

## Methods

### Design

This study was conducted and reported in accordance with the Preferred Reporting Items for Systematic Reviews and Meta-Analyses (PRISMA) statement for reporting systematic reviews and meta-analyses [47]. The systematic review protocol was registered in the International Prospective Register of Systematic Reviews (PROSPERO, registration number: CRD42019119725).

### Search Strategy

We conducted a comprehensive literature search in the following six electronic databases from database inception to September 13, 2019: PubMed, EMBASE, PsycINFO, CINAHL, Web of Science, and Cochrane Central Register of Controlled Trials (CENTRAL). An information specialist was consulted for the search. The search strategy included combinations of relevant medical subject headings and text-based search terms covering computerized (or internet, digital, eHealth, online, smartphone, or web-based), CBT (or cognitive, behavior, therapy, treatment, or intervention), and adolescent (or child, young person, teenager, or youth). The complete search strings are documented in Multimedia Appendix 1. In addition, we manually searched reference lists of included studies and relevant previous reviews, and searched international trial registers for eligible studies, which resulted in one additional record.

### Study Selection

We included RCTs in which computer-based, internet-based, or smartphone-based cognitive behavioral therapy targeting anxiety, depression, or both was compared to an active treatment control condition or passive control condition. The study population involved adolescents or young adults with a mean age between 12 and 25 years and elevated symptoms of anxiety or depressive disorder (ie, either a formal diagnosis or an elevated score on a standardized self-report measure representing at least a mild-to-moderate symptom level). We only included studies with an English abstract available, those that were published in peer-reviewed journals or were PhD theses, and those that contained outcome data on a continuous anxiety or depressive symptom measure that allowed for calculation of effect sizes. If effect sizes could not be calculated, authors were contacted to retrieve the necessary information.

The intervention needed to be primarily delivered via technology (ie, computer, internet, or smartphone). Interventions were categorized as CBT if (1) they were explicitly described as such by the authors of the article and we found no reason to disagree or (2) all authors of this review agreed that the description of the main intervention components could be regarded as CBT. The control condition was defined as active treatment control (ie, face-to-face CBT or treatment as usual [TAU]) or passive control (ie, waiting list/no treatment or information control). Studies in which the control condition involved an active self-help website (ie, including both psychoeducation and exercises) focused specifically on anxiety or depression were excluded. Studies in which the control condition involved a monitoring control website that did not include active self-help content were included. Comorbid psychiatric or medical disorders were not used as an exclusion criterion.

Two authors (CC and MS) conducted the study selection in a stepwise manner. First, titles and abstracts of all studies were independently screened for potential eligibility. Any disagreements were discussed until consensus was reached. Second, the full papers of all included abstracts were independently screened according to the inclusion and exclusion criteria. In case of discrepancy or uncertainty regarding inclusion, a third author (MB) was consulted until consensus was reached.

### Data Extraction

Information on study characteristics, participant characteristics, and mental health outcomes was extracted from each study and included in an Excel spreadsheet. Data extraction was conducted by one reviewer (CC) and checked by a second reviewer (MS). Study characteristics included authors, country, year of publication, study design, recruitment setting (ie, clinical, general population, or schools), inclusion and exclusion criteria, primary outcome measures, and descriptions of the experimental intervention and comparator, including focus of the intervention, information on guidance, number of treatment modules, and adherence rates. Participant characteristics included sample size, mean age, gender, primary diagnostic type (ie, anxiety, depression, or both; either based on diagnosis or an elevated symptom level), and baseline symptom levels. Means and SDs of the outcome measures of anxiety and depressive symptoms at posttreatment assessment were extracted. If available, means and SDs at short-term follow-up (ie, 1-5 months) and long-term follow-up (ie, 6-12 months) were extracted as well.

If possible, we utilized effect sizes of the intention-to-treat sample; if these were not available, we used effect sizes of the completer sample. In case of multiple outcome measures of anxiety and depressive symptoms, we selected the primary outcome measure as stated by the authors. If the authors did not specify any primary outcome measure of anxiety or depressive symptoms, we selected a well-validated and widely-used outcome measure of these symptoms that was used at every time point of the study (ie, both at posttest and follow-up, if applicable). If both an active treatment control and passive control were utilized in a single RCT, outcomes from both conditions were extracted. Our main meta-analyses were conducted separately for active treatment control (ie, face-to-face CBT or face-to-face TAU) and passive control (ie, waiting list, information control, or no treatment). In our subgroup analyses, data from all control groups per study were included. As the inclusion of multiple comparisons of one study in a meta-analysis violates the assumption of independence, we divided the *n* of the intervention group evenly across comparators, which is a procedure recommended by the Cochrane guidelines [48].

### Quality Assessment

The quality of each included study was assessed following the guidelines provided by the Cochrane risk-of-bias tool for randomized trials, version 2.0 (RoB 2) [49]. Risk of bias was examined in the following five domains: (1) bias arising from the randomization process; (2) bias due to deviations from intended interventions; (3) missing outcome data; (4) bias in measurement of the outcome; and (5) bias in selection of the reported result. Each domain was rated as either low risk of bias, some concerns, or high risk of bias. A total score was calculated for each study by adding up the following values for each domain: “0” for low risk of bias, “1” for some concerns, and “2” for high risk of bias.

The overall quality of the evidence for each outcome was assessed using the Grading of Recommendations Assessment, Development and Evaluation (GRADE) approach [50]. The quality of each outcome was assessed for the following domains: (1) risk of bias; (2) inconsistency of results (ie, heterogeneity); (3) indirectness of evidence; (4) imprecision of results; and (5) suspected publication bias. In case of limitations in one domain, the evidence for each outcome was downgraded by one or two levels. Subsequently, the overall evidence for each outcome across domains was categorized as high, moderate, low, or very low, representing the level of certainty of the effect estimates. Both RoB 2 and GRADE assessments were conducted independently by two reviewers (CC and MS), and any disagreements were resolved by discussion until consensus was reached. Cohen κ coefficients were calculated to determine the interrater reliability.

### Statistical Analysis

A random-effects meta-analysis was conducted with the Comprehensive Meta-Analysis software (CMA version 3), using the SMD to calculate pooled mean effect sizes (Hedges *g*). Effect sizes were calculated by subtracting the mean posttest score of the treatment group from the mean score of the comparison group, and dividing the result by the pooled standard deviation of the two groups. Posttreatment means and SDs were compared between the intervention and control groups. Effect sizes of 0.2, 0.5, and 0.8 are considered to be small, medium, and large, respectively [51]. In addition, we calculated the number needed to treat (NNT), using the Kraemer & Kupfer [52] formula. The NNT indicates the total number of patients who need to be treated in order to achieve one additional positive outcome [53].

Heterogeneity was assessed by calculating the *I^2^* statistic, which indicates how much overall variance should be attributed to between-study variance, with a value of 25% representing low heterogeneity, 50% representing moderate heterogeneity, and 75% representing high heterogeneity. In addition, we calculated the 95% CIs around *I^2^* by using the noncentral chi-square approach in the “heterogi” module of STATA [54,55].

Subgroup analyses were conducted to examine the influence on the difference between intervention and control conditions for (1) the diagnostic focus of the intervention (ie, anxiety, depression, or both); (2) age group (ie, adolescents with mean age ≤18 years or young adults with mean age >18 years); (3) the level of guidance (ie, guided or self-guided); (4) the adherence rate, defined as the percentage of participants in the intervention group who completed all treatment modules at posttreatment (ie, low, defined as ≤50%, or high, defined as >50%); (5) recruitment type (ie, clinical, community, or university/school); and (6) the number of treatment modules (ie, <5, 5-9, or 10-14 modules). Subgroup analyses were conducted across studies with interventions focused on anxiety, depression, or both, with multiple control groups per study included. Subgroup analyses were conducted using the mixed-effects model, in which the effect sizes within the subgroups are pooled with the random-effects model, whereas the fixed-effects model is used to test for significant differences between the subgroups. Subgroup analyses involving age, guidance level, and adherence rates were planned a priori based on the literature. In addition, a subgroup analysis on the diagnostic focus of the intervention was planned a priori to test whether it was justified to conduct all subgroup analyses on the total set of studies, including interventions aimed at anxiety, depression, or both. Recruitment type and number of treatment modules were examined post-hoc as these reflect potential sources of heterogeneity. For all six subgroup analyses, a Bonferroni-corrected α level of *P*<.008 was used to account for multiple testing. In addition, bivariate meta-regression analyses were conducted to explore the associations of the mean age of study participants, adherence rate, and risk of bias with effect sizes. Analyses with age and adherence rate were planned a priori, whereas risk of bias level was included post-hoc.

Publication bias was examined as follows. First, the funnel plot of effect sizes was visually inspected. Second, the Duval and Tweedie trim and fill procedure was used to calculate an adjusted pooled effect size that accounts for missing studies due to publication bias [56]. Third, the Egger test was used to quantify the bias captured by the funnel plot [57]. In accordance with the Cochrane guidelines [48], publication bias was only examined in meta-analyses with at least 10 studies.

## Results

### Systematic Review

#### Included Studies

**Figure 1:**
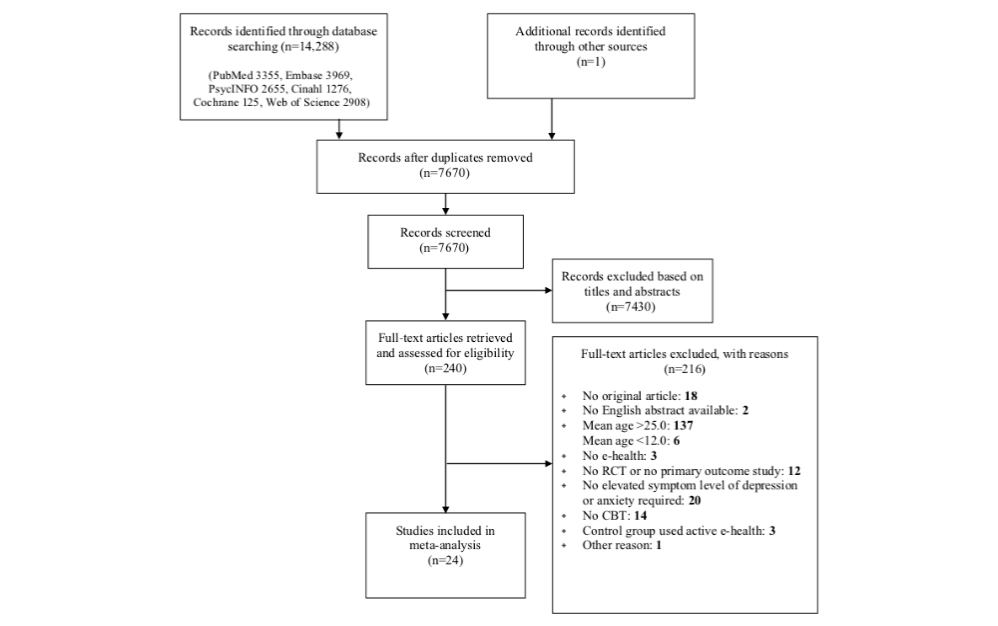
Flow chart.

The database search resulted in 7670 articles, of which we retrieved the full text of 240 articles. Twenty-four studies met all inclusion criteria and were included in the systematic review and meta-analysis (Figure 1). Interrater agreement of inclusion was strong (98.3%; Cohen κ=0.90, *P*<.001). The included studies were published between 2009 and September 2019. Most were conducted in Australia (n=5) and the United Kingdom (n=4). All studies reported posttreatment effects, whereas short-term and long-term follow-up data of both intervention and control conditions were only reported in three and five studies, respectively. The sample sizes of the RCTs ranged from 19 to 257 (mean 92.75, median 70). Twelve studies were primarily aimed at adolescents (age 12-19 years), eight studies were aimed at young adults (age 19-25 years), and four studies had a mixed sample. The mean age varied between 13.31 [58] and 24.40 years [59]. Most studies were conducted in samples of university students (n=8) or community samples (n=7), whereas four studies were conducted in clinical samples, four studies in secondary schools or educational programs, and one study in a mixed sample [60]. Studies targeted participants with a diagnosis or elevated symptoms of depressive disorder (n=10), participants with a diagnosis or elevated symptoms of anxiety disorder (n=8), or participants with elevated symptoms of depressive and/or anxiety disorder (n=6). In total, 19 studies compared cCBT to a waiting list or no treatment control condition, of which five studies also included a face-to-face CBT control condition. Four studies compared cCBT to a placebo condition (information control or attention control), and one study compared cCBT to TAU. Selected characteristics of the included studies are presented in Multimedia Appendix 2.

#### Description of the Interventions

Seventeen studies investigated cCBT programs delivered via the internet (also known as iCBT), such as MoodGym [60-62] and BRAVE [63,64]. Of these interventions, most were completed at the respondent’s home (n=12), whereas five were completed at a treatment or research site. Seven studies investigated cCBT programs delivered via a computer program or CD-ROM, such as SPARX [65-67], Woebot [68], and Stressbusters [58]. Of these interventions, four were completed at the respondent’s home (n=4) and three were completed at school or a treatment site. The regular treatment components of cCBT were psychoeducation, behavioral activation, cognitive restructuring, exposure, problem-solving, and homework assignments.

#### Treatment Duration and Intensity

The number of treatment modules ranged from 3 to 12 in 21 included studies (mean 7.1, median 7). Two studies did not report the exact number of modules [59,69]. One study examined the Woebot intervention [68], which does not consist of different modules, but delivers cCBT by 1 to 20 (median 12) automated conversations and mood tracking in an instant messenger app. The 24 included interventions were completed over a period of 2 to 16 weeks (mean 7.5, median 7).

#### Guidance

In 14 studies, participants were guided through the intervention by a therapist or researcher. The other 10 interventions were self-guided (ie, unguided) [58,59,65-72]. Guidance was provided through telephone and/or email contact [63,64,73-78], chat sessions [79], or face-to-face guidance during the participant’s completion of the modules [60-62,80,81]. In general, guidance consisted of monitoring progress and providing support, encouragement, and clarification. In nine studies, guidance additionally included providing personalized feedback on completed exercises [63,64,74-80].

#### Adherence

Adherence rates were reported in 19 studies. Only 10 studies reported the most common measure of adherence (ie, the number of completed sessions divided by the maximum number of sessions). In these studies, adherence ranged from 32.2% to 100% (mean 76.91%, median 78%). An alternative measure of adherence, namely the percentage of participants in the intervention group who completed all treatment modules, was reported in 19 studies, with adherence rates ranging from 0% to 100% (mean 57.12%, median 60%)*.* Adherence rates for each study are presented in Multimedia Appendix 3.

#### Quality of Evidence

**Figure 2:**
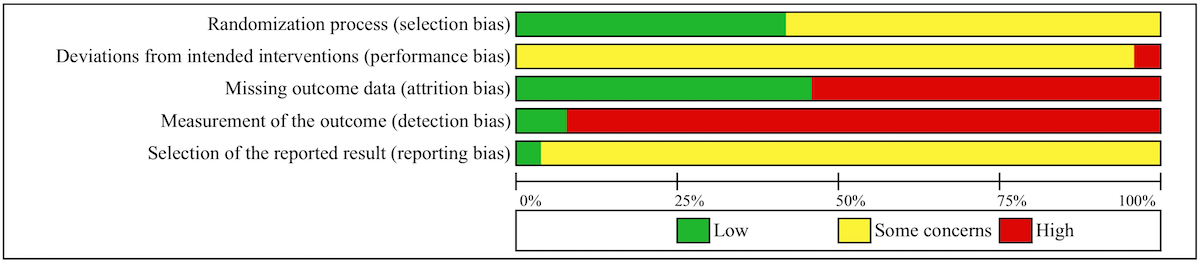
Risk of bias graph.

Based on the Cochrane RoB 2 tool [49], 22 out of 24 studies had an overall high risk of bias, and the remaining two studies were rated as “some concerns.” The overall high risk of bias in this vast majority of included studies was mainly due to an increased risk of bias in the measurement of the outcome, caused by the use of self-report measures or the unblinded use of observer-rated measures. Furthermore, 13 out of 24 studies also had an increased risk of bias due to missing outcome data, since >5% of their data were missing and no sufficiently appropriate analysis (eg, multiple imputation) was used to handle the missing data. Lastly, one study was rated as having an increased risk of bias due to deviations from the intended intervention [77]. Interrater reliability for the risk of bias was very good (κ=0.89, *P*<.001). Figure 2 demonstrates the authors’ conclusions regarding the risk of bias across studies. Multimedia Appendix 4 presents the risk of bias classifications per domain assigned to each study. It should be noted that self-report measures are widely used in psychological treatment studies, especially in studies on computerized treatment. Therefore, the current rating may be too strict. Without taking into account the risk of bias in the measurement of the outcome, 13 out of 24 studies had an overall high risk of bias.

The overall quality of the evidence for each outcome was assessed using the GRADE approach [50]. The quality rate for each outcome is shown in Tables 1-3. In summary, although the overall quality of some outcomes was moderate, the overall quality of most outcomes was low. Since almost all studies were associated with a high risk of bias based on the RoB tool, all outcomes were downgraded one level for this domain. Many outcomes were downgraded one additional level for inconsistency, because of substantial heterogeneity in the meta-analysis. One outcome was downgraded one additional level for imprecision of results due to a small sample size. Interrater reliability for the quality of evidence was very good (κ=0.87, *P*<.001).

**Table 1:** Effect sizes regarding depressive symptoms in the meta-analysis of studies comparing computerized cognitive behavior therapy in adolescents and young adults with active treatment and passive controls at posttreatment.

Variable			N_com_^a^	N_par_^b^	Effect size		Heterogeneity		Grade^c^	NNT^d^
					*g*	95% CI	*I* ^2^	95% CI		
**All studies**										
	Active treatment controls		5	403	−0.55	−1.18 to 0.08	87.52	73 to 94	++	3.31
	**Passive controls**		20	1604	0.52^e^	0.33 to 0.71	68.69	50 to 80	++	3.5
		One outlier removed^f^	19	1558	0.46^e^	0.29 to 0.63	58.49	31 to 75	+++	3.91
**Studies aimed at depression**										
	Active treatment controls		4	351	−0.70	−1.51 to 0.11	90.63	79 to 96	+	2.63
	**Passive controls**		13	1162	0.60^e^	0.35 to 0.85	73.27	54 to 85	++	3.05
		One outlier removed^f^	12	1116	0.51^e^	0.30 to 0.72	61.76	28 to 80	++	3.55

^a^N_com_: number of comparisons.

^b^N_par_: number of participants.

^c^+: very low quality; ++: low quality; +++: moderate quality.

^d^NNT: number needed to treat.

^e^*P*<.001.

^f^Outlier Sethi (2013) excluded.

**Table 2:** Effect sizes regarding depressive symptoms in the subgroup analyses of studies comparing computerized cognitive behavior therapy in adolescents and young adults with active treatment and passive controls at posttreatment.

Variable		N_com_^a^	N_par_^b^	Effect size		Heterogeneity		*P*	NNT^c^
				*g*	95% CI	*I* ^2^	95% CI		
**Diagnostic focus^d^**								.63	
	Anxiety	8	464	0.32^e^	0.04 to 0.60	50.78	0 to 78		5.56
	Depression	9	1152	0.44^f^	0.21 to 0.67	75.44	53 to 87		4.1
	Both	4	182	0.55^e^	0.13 to 0.96	0	0 to 85		3.31
**Age group^d^**								.29	
	Adolescents	12	1027	0.34^g^	0.14 to 0.54	61.51	28 to 79		5.26
	Young adults	9	771	0.50^f^	0.28 to 0.73	39.7	0 to 72		3.62
**Guidance^d^**								.56	
	Guided	10	771	0.46^f^	0.23 to 0.70	56.96	13 to 79		3.91
	Self-guided	11	1027	0.37^g^	0.16 to 0.58	58.97	20 to 79		4.85
**Adherence^d^**								.77	
	Low	6	779	0.39^e^	0.09 to 0.69	77.56	50 to 90		4.59
	High	10	737	0.48^f^	0.23 to 0.72	62.06	25 to 81		3.76
**Recruitment type^d^**								.53	
	Clinical	4	376	0.24	−0.11 to 0.59	0	0 to 85		7.46
	Community	5	462	0.50^g^	0.18 to 0.82	78.12	47 to 91		3.62
	University/school	12	960	0.44^f^	0.23 to 0.65	50.27	3 to 74		4.1
**Number of modules^d^**								.21	
	<5	3	138	0.65^g^	0.20 to 1.11	0	0 to 90		2.82
	5-9	12	1097	0.49^f^	0.29 to 0.69	69.11	44 to 83		3.68
	10-14	3	377	0.14	−0.23 to 0.52	0	0 to 90		12.82

^a^N_com_: number of comparisons.

^b^N_par_: number of participants.

^c^NNT: number needed to treat.

^d^Outliers Sethi (2010) and Sethi (2013) excluded.

^e^*P*<.05.

^f^*P*<.001.

^g^*P*<.01.

**Table 3:** Bivariate meta-regression analyses regarding depressive symptoms in studies comparing computerized cognitive behavior therapy in adolescents and young adults with active treatment and passive controls at posttreatment.

Variable		N_com_^a^	*b*	95% CI	*P*
**Mean age^b^**					
	Intercept	21	0.14	−0.55 to 0.84	.69
	Mean age	21	0.01	−0.02 to 0.05	.44
**Adherence^b^**					
	Intercept	16	0.39	−0.02 to 0.79	.06
	Adherence	16	0.01	−0.01 to 0.01	.75
**Risk of bias^b^**					
	Intercept	21	−0.07	−0.68 to 0.53	.81
	Risk of bias	21	0.11	−0.01 to 0.23	.08

^a^N_com_: number of comparisons.

^b^Outliers Sethi (2010) and Sethi (2013) excluded.

### Meta-Analysis

#### Effects of cCBT on Depressive Symptoms at Posttreatment

The pooled effect size of cCBT for depressive disorders, anxiety disorders, or both regarding depressive symptoms at posttreatment compared with active treatment controls was *g*=−0.55 (95% CI −1.18 to 0.08, *P*=.09, *k*=5; Table 1), and heterogeneity was high (*I^2^*=87.52%, 95% CI 73-94). Compared with passive controls, the pooled effect size of cCBT was *g*=0.52 (95% CI 0.33-0.71, *P*<.001, *k*=20), and heterogeneity was moderate (*I^2^*=68.69%, 95% CI 50-80). Removing one extreme outlier with an effect size of *g*=1.93 [60] resulted in a somewhat smaller mean effect size of *g*=0.46 (95% CI 0.29-0.63, *P*<.001, *k*=19), with a lower, though still moderate, heterogeneity (*I^2^*=58.49%, 95% CI 31-75).

In studies aimed specifically at depressive disorders or both depressive and anxiety disorders, the nonsignificant pooled effect size of cCBT compared with active treatment controls regarding depressive symptoms was *g*=−0.70 (95% CI −1.51 to 0.11, *P*=.09, *k*=4), corresponding to an NNT of 2.63 in favor of active treatment controls. Heterogeneity was very high (*I^2^*=90.63%, 95% CI 79-96). When compared with passive controls, cCBT yielded a significant medium effect size of *g*=0.60 (95% CI 0.35-0.85, *P*<.001, *k*=13), corresponding to an NNT of 3.05. Heterogeneity was moderate to high (*I^2^*=73.27, 95% CI 54-85). Removing the extreme outlier [60] again resulted in a somewhat smaller mean effect size of *g*=0.51 (95% CI 0.30-0.72, *P*<.001, *k*=12), with a lower, though still moderate, heterogeneity (*I^2^*=61.76, 95% CI 28-80) and a corresponding NNT of 3.55. Inspection of the funnel plot and the Duval and Tweedie trim and fill procedure showed no indication of publication bias, and the Egger test of the intercept was not significant (*P*=.74), indicating no need to adjust for missing studies. Multimedia Appendix 5 and Multimedia Appendix 6 provide forest plots of effect sizes regarding depressive symptoms for active treatment controls and passive controls, respectively.

A series of subgroup analyses (Table 2) was conducted across studies focused on depression, anxiety, or both, with multiple control groups per study included and two extreme outliers excluded [60,62]. Heterogeneity remained moderate in most subgroups. Effects in all but two subgroups were significantly different from zero, and all were in favor of cCBT. We found no indication that the diagnostic focus of the intervention, age group, level of guidance, adherence rate, type of recruitment, or number of treatment modules was associated with differential effect sizes. Lastly, bivariate meta-regression analyses (Table 3) showed no significant association of the mean age of study participants (*b*=0.01; 95% CI −0.02 to 0.05, *P*=.44), adherence (*b*=0.01; 95% CI −0.01 to 0.01, *P*=.74), or risk of bias (*b*=0.11; 95% CI −0.01 to 0.23, *P*=.08) with effect size regarding depressive symptoms.

#### Effects of cCBT on Anxiety Symptoms at Posttreatment

Regarding anxiety symptoms at posttreatment, the pooled effect size of cCBT for anxiety disorders, depressive disorders, or both compared with active treatment controls was *g*=0.06 (95% CI −0.13 to 0.26, *P=*.53, *k*=5; Table 4). Heterogeneity was low, although the wide 95% CI indicated some uncertainty regarding the exact level of heterogeneity (*I^2^*=0.00%, 95% CI 0-79). Compared with passive controls, cCBT yielded a significant pooled effect size of *g*=0.49 (95% CI 0.29-0.68, *P*<.001, *k*=21), and heterogeneity was moderate (*I^2^*=68.17%, 95% CI 50-80). Removing one extreme outlier with an effect size of *g*=1.94 [60] resulted in a slightly smaller mean effect size of *g*=0.42 (95% CI 0.25-0.59, *P*<.001, *k*=20), with a lower, though still moderate, heterogeneity (*I^2^*=57.42%, 95% CI 30-74).

When only including studies with cCBT aimed specifically at anxiety disorders or at both anxiety and depressive disorders, the nonsignificant pooled effect size regarding anxiety symptoms compared with active treatment controls remained similar (*g*=0.04, 95% CI −0.23 to 0.31, *P*=.79, *k*=4). Heterogeneity was low, although the wide 95% CI again indicated uncertainty regarding the exact level of heterogeneity (*I^2^*=0.00%, 95% CI 0-85). Compared with passive controls, cCBT yielded a pooled effect size of *g*=0.59 (95% CI 0.34-0.84, *P*<.001, *k*=16), corresponding to an NNT of 3.09, and heterogeneity was moderate (*I^2^*=67.83%, 95% CI 46-81). Again, removing the extreme outlier [60] resulted in a smaller mean effect size of *g*=0.50 (95% CI 0.29-0.71, *P*<.001, *k*=15), with an NNT of 3.62 and a lower, though still moderate, heterogeneity (*I^2^*=52.57%, 95% CI 15-74). Inspection of the funnel plot and the Duval and Tweedie trim and fill procedure showed a minor indication of publication bias, but the Egger test of the intercept was not significant (*P*=.11). Adjusting for missing studies using the Duval and Tweedie trim and fill procedure resulted in a slightly smaller overall effect size of *g*=0.44 (95% CI 0.23-0.65), corresponding to an NNT of 4.10. Multimedia Appendix 7 and Multimedia Appendix 8 provide forest plots of effect sizes regarding anxiety symptoms for active treatment and passive control conditions, respectively.

A series of subgroup analyses were conducted across studies focused on anxiety, depression, or both, with multiple comparators per study included and one outlier excluded [60]. Heterogeneity was moderate in most subgroups (Table 5). Effects in most subgroups were different from zero, and all were in favor of cCBT. We found no indication that the diagnostic focus of the intervention, age group, type of guidance, adherence rate, type of recruitment, or number of treatment modules was associated with differential effect sizes. Lastly, bivariate meta-regression analyses (Table 6) showed no significant association of the mean age of study participants (*b*=0.02; 95% CI −0.01 to 0.06, *P*=.21), adherence (*b*=0.00; 95% CI −0.01 to 0.00, *P*=.65), or risk of bias (*b*=0.04; 95% CI −0.05 to 0.13, *P*=.36) with effect size regarding anxiety symptoms.

**Table 4:** Effect sizes regarding anxiety symptoms in the meta-analysis of studies comparing computerized cognitive behavior therapy in adolescents and young adults with active treatment and passive controls at posttreatment.

Variable				N_com_^a^		N_par_^b^		Effect size				Heterogeneity				Grade^c^		NNT^d^
								*g*		95% CI		*I* ^2^		95% CI				
**All studies**																		
	Active treatment controls			5		390		0.06		−0.13 to 0.26		0		0 to 79		++		29.41
	**Passive controls**			21		1570		0.49^e^		0.29 to 0.68		68.17		50 to 80		++		3.68
		One outlier removed^f^	20		1524		0.42^e^		0.25 to 0.59		57.42		30 to 74		+++		4.27	
**Studies aimed at anxiety**																		
	Active treatment controls			4		203		0.04		−0.23 to 0.31		0		0 to 85		++		45.45
	**Passive controls**			16		868		0.59^e^		0.34 to 0.84		67.83		46 to 81		++		3.09
		One outlier removed^f^	15		822		0.50^e^		0.29 to 0.71		52.57		15 to 74		+++		3.62	
		Trim and fill adjusted values					0.44		0.23 to 0.65								4.1	

^a^N_com_: number of comparisons.

^b^N_par_: number of participants.

^c^++: low quality; +++: moderate quality.

^d^NNT: number needed to treat.

^e^*P*<.001.

^f^Outlier Sethi (2013) excluded.

**Table 5:** Effect sizes regarding anxiety symptoms in the subgroup analyses of studies comparing computerized cognitive behavior therapy in adolescents and young adults with active treatment and passive controls at posttreatment.

Variable		N_com_^a^	N_par_^b^	Effect size		Heterogeneity		*P*	NNT^c^
				*g*	95% CI	*I* ^2^	95% CI		
**Diagnostic focus^d^**									
	Anxiety	12	687	0.47^e^	0.25 to 0.69	34.28	0 to 67	.39	3.85
	Depression	6	889	0.23	−0.03 to 0.50	71.68	34 to 88		7.69
	Both	6	211	0.33	−0.04 to 0.69	61.89	7 to 84		5.43
**Age group^d^**									
	Adolescents	13	1031	0.25^f^	0.06 to 0.44	44.27	0 to 71	.08	7.14
	Young adults	11	756	0.51^e^	0.29 to 0.73	51.47	3 to 76		3.55
**Guidance^d^**									
	Guided	15	958	0.41^e^	0.21 to 0.61	48.01	5 to 71	.47	4.39
	Self-guided	9	829	0.30^g^	0.07 to 0.53	60.28	17 to 81		5.95
**Adherence^d^**									
	Low	8	894	0.44^e^	0.19 to 0.68	38.77	0 to 73	.61	4.1
	High	11	655	0.27^g^	0.04 to 0.51	55.6	13 to 77		6.58
**Recruitment type^d^**									
	Clinical	3	267	0.06	−0.34 to 0.46	0	0 to 90	.28	29.41
	Community	8	620	0.43^f^	0.17 to 0.68	58.97	10 to 81		4.2
	University/school	13	900	0.40^e^	0.20 to 0.61	51.22	7 to 74		4.5
**Number of modules^d^**									
	<5	5	167	0.47^g^	0.05 to 0.89	68.76	20 to 88	.91	3.85
	5-9	12	1051	0.38^f^	0.16 to 0.60	63.37	32 to 80		4.72
	10-14	5	492	0.29	−0.04 to 0.61	12.58	0 to 82		6.17

^a^N_com_: number of comparisons.

^b^N_par_: number of participants.

^c^NNT: number needed to treat.

^d^Outlier Sethi (2013) excluded.

^e^*P*<.001.

^f^*P*<.01.

^g^*P*<.05.

**Table 6:** Bivariate meta-regression analyses regarding anxiety symptoms in studies comparing computerized cognitive behavior therapy in adolescents and young adults with active treatment and passive controls at posttreatment.

Variable		N_com_^a^	*b*	95% CI	*P*
**Mean age^b^**					
	Intercept	24	−0.07	−0.77 to 0.63	.84
	Mean age	24	0.02	−0.01 to 0.06	.21
**Adherence^b^**					
	Intercept	19	0.42	0.10 to 0.74	.01
	Adherence	19	0.00	−0.01 to 0.00	.65
**Risk of Bias^b^**					
	Intercept	24	0.13	−0.38 to 0.65	.61
	Risk of Bias	24	0.04	−0.05 to 0.13	.36

^a^N_com_: number of comparisons.

^b^Outlier Sethi (2013) excluded.

#### Short-Term Follow-Up Effects

Three studies reported short-term follow-up effects (ie, up to 5 months posttreatment) for cCBT on depressive symptoms. The pooled effect size for studies with cCBT aimed specifically at depressive disorders or at both depressive and anxiety disorders compared with active treatment controls was not significant (*g*=0.12, 95% CI −0.11 to 0.35, *P*=.29, *k*=2; Table 7). Compared with passive controls, the pooled effect size showed no significant difference between cCBT and control conditions either (*g*=0.19, 95% CI −0.08 to 0.46, *P*=.16, *k*=2). Although effect sizes were in favor of cCBT, these results indicated that cCBT is not superior to controls at short-term follow-up. However, owing to the small number of comparisons, the statistical power to detect small differences was limited. Heterogeneity was low (*I^2^*=0.00%), but the number of studies was too small to enable calculation of 95% CI. No studies reported short-term follow-up effects for cCBT on anxiety symptoms.

**Table 7:** Effect sizes regarding depressive and anxiety symptoms in the meta-analysis of studies comparing computerized cognitive behavior therapy in adolescents and young adults with active treatment and passive controls at short-term follow-up (1-5 months) and long-term follow-up (6-12 months).

Variable			N_com_^a^	N_par_^b^	Effect size		Heterogeneity		Grade^c^	NNT^d^
					*g*	95% CI	*I* ^2^	95% CI		
**Depressive symptoms**										
	**Short-term follow-up**									
		Active treatment controls	2	288	0.12	−0.11 to 0.35	0	N/A^e^	+++	14.71
		Passive controls	2	211	0.19	−0.08 to 0.46	0	N/A^e^	++	9.43
	**Long-term follow-up^f^**									
		Passive controls	3	461	0.27^g^	0.09 to 0.45	0	0 to 90	+++	6.58
**Anxiety symptoms**										
	**Long-term follow-up^h^**									
		Active treatment controls	2	140	0.08	−0.41 to 0.56	50.61	N/A^e^	++	21.74

^a^N_com_: number of comparisons.

^b^N_par_: number of participants.

^c^++: low quality; +++: moderate quality.

^d^NNT: number needed to treat.

^e^N/A: not applicable; calculation of 95% CI not possible because *df*=1.

^f^Only one study with active treatment controls available.

^g^*P*<.01.

^h^No studies with passive controls available.

#### Long-Term Follow-Up Effects

Three studies reported long-term follow-up effects (ie, 6-12 months posttreatment) for cCBT on depressive symptoms. The pooled effect size indicated cCBT aimed at depressive symptoms or both depressive and anxiety symptoms to be effective compared with passive controls at long-term follow-up (*g*=0.27, 95% CI 0.09-0.45, *P*=.004, *k*=3), corresponding with an NNT of 6.58. Heterogeneity was low, although the wide 95% CI indicated uncertainty regarding the exact level of heterogeneity (*I^2^*=0.00%, 95% CI 0-90). As only one study [65] reported long-term follow-up effects for cCBT on depressive symptoms compared with active treatment controls, meta-analysis was not possible.

Only two studies reported long-term follow-up effects (ie, 6-12 months posttreatment) for cCBT aimed at anxiety or both anxiety and depression on anxiety symptoms. The pooled effect size showed no significant effect for cCBT compared with active treatment controls (*g*=0.08, 95% CI −0.41 to 0.56, *P*=.75, *k*=2) at long-term follow-up. No study reported long-term follow-up effects for cCBT on anxiety symptoms compared with passive controls.

## Discussion

### Principal Findings

This study provides an up-to-date meta-analysis examining the effects of cCBT on anxiety and depressive symptoms in adolescents and young adults compared with active treatment and passive controls, differentiating between posttreatment and follow-up. Our results indicate that cCBT is beneficial for reducing anxiety and depressive symptoms at posttreatment in adolescents and young adults compared with passive controls, with small to medium effect sizes. For cCBT aimed at depressive disorders or depressive and anxiety disorders, we found a pooled effect size of *g*=0.51 regarding depressive symptoms, which corresponds to an NNT of 3.55. For cCBT aimed at anxiety disorders or anxiety and depressive disorders, we found an effect size of *g*=0.50 regarding anxiety symptoms. After adjustment for missing studies owing to a minor indication of publication bias, the effect size lowered slightly to *g*=0.44, corresponding to an NNT of 4.10. Compared with active treatment controls, the pooled effect size regarding depressive symptoms was in favor of controls (*g*=−0.70). However, the effect size was not significant and heterogeneity was very high. For anxiety symptoms, cCBT and active treatment controls showed similar effects (*g*=0.04). Subgroup analyses did not reveal any differences between groups; however, owing to the small number of studies, the statistical power to detect small differences was limited. Meta-regression analyses showed no associations between age, adherence rate, or risk of bias and effect sizes.

Overall, this study shows robust evidence of the effectiveness of cCBT in reducing anxiety and depressive symptoms in adolescents and young adults compared with passive controls. Our results are largely in line with those of previous studies on cCBT in children and young people aged up to 25 years [36], adolescents and young adults aged 12 to 25 years [37] and 10 to 25 years [43], and children and adolescents aged up to 18 years [44]. However, these studies generally reported somewhat larger effect sizes (range 0.62-0.77) regarding both depressive symptoms and anxiety symptoms [36], anxiety symptoms [37], or depressive and/or anxiety symptoms [44] compared with passive controls [36,37,44]. Similarly, research in adults found larger effect sizes for cCBT regarding depression and anxiety compared with passive controls (*g*=0.90) [34]. Compared with the effects of traditional face-to-face CBT in children and adolescents with anxiety disorders against waiting list controls (NNT=3.0) [19], we found a somewhat lower NNT for cCBT against passive controls (NNT=4.10) after adjusting for potential publication bias. Effect sizes in our study were similar to those found in a meta-analysis comparing face-to-face CBT for depression (0.60) and anxiety disorders (0.48) to passive controls in college and university students [21].

Our results suggest that the effects of cCBT do not differ from those of active treatment controls (ie, face-to-face CBT or face-to-face TAU) regarding anxiety symptoms, but may be inferior to active treatment controls regarding depressive symptoms, although the effect size was not significant. These findings are in line with those of previous meta-analyses in youth across three early studies that were also included in the current meta-analysis [37,41]. Although our meta-analysis included three additional studies, the number of RCTs comparing cCBT with face-to-face treatment in adolescents and young adults remains small. With regard to depressive symptoms, heterogeneity was very high (*I^2^*=90.63), and the pooled effect size should be interpreted with caution. Hence, more research directly comparing both treatments is needed to determine whether cCBT is effective compared with face-to-face treatment controls in adolescents and young adults. Research in adults has shown largely equivalent effects of cCBT on both anxiety and depressive symptoms compared with face-to-face CBT [34,35]. However, the number of studies directly comparing cCBT with face-to-face CBT in adults remains limited as well.

This study also aimed to investigate the effectiveness of cCBT at short-term and long-term follow-ups. However, the number of studies reporting follow-up effects was limited. Regarding long-term effects, cCBT was effective in reducing depressive symptoms compared with passive controls, with a small effect size (*g*=0.27). Our results indicated no long-term follow-up effect for cCBT on anxiety symptoms compared with active treatment controls. Meta-analyses of short-term follow-up data on depressive symptoms indicated similar effects for cCBT compared with active treatment and passive controls. Only Välimäki et al [43] investigated the follow-up effects of cCBT and other internet-based interventions in adolescents and young adults, reporting mid-term and long-term effects on depressive symptoms and mid-term effects on anxiety symptoms. However, they only reported mean differences, and no standardized effect size or NNT. In addition, they did not separately examine the effects of cCBT. Moreover, their selection of studies was based on interventions aimed at depression and not anxiety. Therefore, their results are not easily comparable to those of the current study.

Importantly, owing to the small number of studies reporting follow-up effects, the power to detect small effect sizes was limited in both this study and the study of Välimäki et al [43]. In contrast, the number of studies reporting follow-up effects of cCBT in adults is substantially larger. A recent meta-analysis found 29 trials that reported short-term follow-up effects and 15 trials that reported long-term follow-up effects [34]. cCBT for depressive disorder or anxiety disorder was found to be effective at short-term follow-up (ie, 3-6 months) and long-term follow-up (ie, 9-18 months) compared with posttreatment effect sizes, with small effect sizes across disorders (ie, *g*=0.15 and *g*=0.22, respectively). In contrast with the small effect sizes identified in our study, Andersson et al [82] found very large effect sizes for cCBT regarding depressive or anxiety symptoms in adults (*g*=1.31 across 10 studies) at long-term follow-up of 2 to 5 years compared with mainly passive controls. However, the authors noted that it was unclear whether randomization remained intact over the follow-up period. In summary, in order to determine the long-term effects of cCBT in adolescents and young adults, it is of great importance that future studies include follow-up assessments. Studies comparing cCBT with active treatment control conditions should aim to maintain randomization during the entire follow-up period.

Furthermore, this study aimed to explore whether respondents’ age, guidance level, and treatment adherence were associated with effect sizes. No moderators of treatment effects could be identified. We found no differences in effect sizes for adolescents and young adults regarding anxiety or depressive symptoms, and no association between respondents’ mean age and effect sizes. Previous studies that examined the moderating role of age in meta-analyses among youth reported mixed results. Pennant et al [37] found a higher effect size in young adults compared with adolescents regarding anxiety symptoms, but not depressive symptoms. However, the authors noted that these groups also differed in terms of symptom level, which may have caused the difference in effect sizes. Ebert et al [36] and Podina et al [42] found a higher effect size in adolescents compared with children [36], whereas others [38,44] did not find evidence for such a moderating role of age. Regarding the absence of an association between guidance level and effect sizes, our results correspond with those of Pennant et al [37] in the same age groups. Studies in children and adolescents found mixed results, with Podina et al [42] reporting higher effect sizes for lower levels of guidance and Grist et al [44] reporting higher effect sizes for higher levels of guidance. The lack of an association between adherence rates and effect sizes in our study contrasts findings in adults with depression and anxiety [33,83]. However, most studies included in this meta-analysis did not report the most common operationalization of treatment adherence, and several did not report any information on treatment completion. Post-hoc subgroup analyses and meta-regression analyses found no association of the diagnostic focus of the intervention, risk of bias, recruitment type, or number of sessions with effect sizes.

This study included a thorough evaluation of the risk of bias and quality of evidence, which indicated an overall high risk of bias in 22 out of 24 studies, and, accordingly, low to moderate overall quality of evidence. The high risk of bias was mainly due to an increased risk of bias in measurement of the outcome caused by self-report or unblinded use of observer-rated measures. However, as self-report measures allow both treatment and outcome measures of studies on computerized interventions to be completed entirely from the participant’s home, they are commonly used in studies on cCBT. As such, using the Cochrane RoB tool 2.0, a high risk of bias in measurement of the outcome is inevitable in many studies on computerized interventions for depression and anxiety. Nevertheless, studies ideally should aim to complement self-report measures with blinded observer-rated outcomes, although measures of anxiety and depressive symptoms will always remain subjective to some extent, even when observer-rated outcomes are used.

### Limitations

Several limitations should be considered when interpreting the results. First, the number of studies in the meta-analyses was limited, especially with regard to short-term and long-term follow-up effects. Therefore, the power to detect small effect sizes was limited. Likewise, subgroup analyses consisted of a small number of comparisons, and the lack of relevant differences in most subgroup analyses might be caused by low statistical power. Second, the included studies showed large variations in intervention content, treatment intensity, and outcome measures. Heterogeneity was considerable in the majority of analyses, and pooled effect sizes should be interpreted with caution. Third, most studies had a high risk of bias owing to the use of self-report measures and/or inappropriate handling of missing data. Overall quality was low for most comparisons because of the high risk of bias and, in most cases, considerable heterogeneity. Lastly, almost all studies were conducted in high-income countries, and most studies in young adults were conducted among university students. Hence, generalizability of these results to other populations may be limited.

### Future Directions

In the rapidly growing field of computerized mental health treatment in adolescents and young adults, new interventions are developed at a fast pace. Since the publication of the most recent previous meta-analysis in adolescents and young adults [43], six new studies were published, which have been included in our meta-analysis. However, the evidence base in young people remains limited compared with the large body of research in adults, and the quality of RCTs is often low. In addition, most RCTs have compared cCBT to passive control conditions, which appears to lead to an overestimation of effects. It is of utmost importance to compare the effects of cCBT with gold standard face-to-face treatment in order to determine whether cCBT can provide an equally effective alternative. Furthermore, more rigorous high-quality research in accordance with the CONSORT and CONSORT eHealth guidelines for conducting and reporting RCTs [84,85] is needed. In particular, future studies should minimize risk of bias by appropriately handling missing data and, ideally, complementing the use of self-report questionnaires with blinded observer-rated measures. Future research should also include larger sample sizes and longer follow-up periods in which randomization is maintained in case of active control groups [82] and should report adherence rates. Finally, future studies should investigate the effect of cCBT in lower-educated samples, as well as young people from low-income countries, for whom face-to-face mental health treatment is often unavailable [86-88]. When high-quality evidence in adolescents and young adults accumulates, future researchers will be able to draw stronger conclusions on the effectiveness of cCBT compared with both active treatment and passive controls and to determine differences in effect sizes for various subgroups and populations.

### Conclusions

This meta-analysis provides robust evidence for the effectiveness of cCBT in the treatment of anxiety and depressive disorders in adolescents and young adults compared with passive controls, with small to medium posttreatment effect sizes. Furthermore, our results indicate that effects of cCBT are similar to those of active treatment controls in reducing anxiety symptoms. Regarding depressive symptoms, however, the results remain unclear, since heterogeneity was high and the number of studies comparing cCBT with active treatment controls was small. No moderators of treatment effects could be identified. cCBT appears to be a promising treatment option for young people, of whom most do not receive face-to-face treatment [23-25]. Importantly, this study also demonstrates the need for more methodologically high-quality research in this population, including active treatment control groups and long-term follow-up assessments.

## Appendix

Multimedia Appendix 1 Search strings.

Multimedia Appendix 2 Selected characteristics of the included studies examining the effects of computerized cognitive behavior therapy for depression and anxiety in adolescents and young adults.

Multimedia Appendix 3 Adherence rates of the included studies examining the effects of computerized cognitive behavior therapy for depression and anxiety in adolescents and young adults.

Multimedia Appendix 4 Risk of bias classification for each study.

Multimedia Appendix 5 Forest plot with standardized posttreatment effect sizes (Hedges g) regarding depressive symptoms for computerized cognitive behavior therapy compared with active treatment control conditions.

Multimedia Appendix 6 Forest plot with standardized posttreatment effect sizes (Hedges g) regarding depressive symptoms for computerized cognitive behavior therapy compared with passive control conditions.

Multimedia Appendix 7 Forest plot with standardized posttreatment effect sizes (Hedges g) regarding anxiety symptoms for computerized cognitive behavior therapy compared with active treatment control conditions.

Multimedia Appendix 8 Forest plot with standardized posttreatment effect sizes (Hedges g) regarding anxiety symptoms for computerized cognitive behavior therapy compared with passive control conditions.

## Abbreviations

CBT: cognitive behavior therapy
cCBT: computerized cognitive behavior therapy
CMA: Comprehensive Meta-Analysis software
GRADE: Grading of Recommendations Assessment, Development and Evaluation
NNT: number needed to treat
RCT: randomized controlled trial
RoB 2: risk-of-bias tool for randomized trials, version 2.0
SMD: standardized mean difference
TAU: treatment as usual
