# Supplementary material for: Internet and Computer-Based Cognitive Behavioral Therapy for Anxiety and Depression in Adolescents and Young Adults: Systematic Review and Meta-Analysis
Source: J Med Internet Res. 2020 Sep 25;22(9):e17831. doi: 10.2196/17831 (PMC7547394; doi:10.2196/17831)
Supplement: Multimedia Appendix 1 [file jmir_v22i9e17831_app1.docx]

**Multimedia Appendix.**

**Search strings**

**PubMed**

**Depression/Anxiety**“Depressive disorder”[mesh terms] OR “Depression”[mesh terms] OR “Mood Disorders”[mesh terms] OR depress*[tiab] OR dysthymi* [tiab] OR “Anxiety”[mesh terms] OR “Anxiety disorders”[mesh terms] OR anxi*[tiab] OR social anxi*[tiab] OR separation anxi*[tiab] OR phobi* [tiab] OR generalised anxiety disorder* [tiab] OR generalized anxiety disorder*[tiab] OR OCD[tiab] OR obsessive compulsive disorder*[tiab] OR hoarding [tiab]

**Population**“Child”[mesh terms] OR “Adolescent” [mesh terms] OR child [tiab] OR children [tiab] OR childhood [tiab] OR teen [tiab] OR teens [tiab] OR teenager* [tiab] OR adolescen*[tiab] OR young per*[tiab] OR youth*[tiab] OR boy [tiab] OR boys[tiab] OR girl [tiab] OR girls[tiab] OR “Young Adult”[mesh] OR young adult* [tiab] OR juvenile*[tiab] OR young people[tiab] OR youngsters [tiab] OR student*[tiab] OR college [tiab] OR schoolchild*[tiab] OR preadolescen*[tiab] OR junior high*[tiab] OR highschool*[tiab] OR senior high[tiab] OR minors[tiab] OR boyhood [tiab] OR girlhood [tiab]

**E-Health**

Web-assist*[tiab] OR web-deliver* [tiab] OR web-mediated*[tiab] OR web-based[tiab] OR mobile app[tiab] OR telehealth[tiab] OR tele-health[tiab] OR telepsychiatry[tiab] OR tele-psychiatry[tiab] OR tele-therap*[tiab] OR teletherap*[tiab] OR tele-medicine[tiab] OR telemedicine[tiab] OR telecare[tiab] OR tele-care[tiab] OR computer-based*[tiab] OR computer-deliver* [tiab] OR computer-assist*[tiab] OR computer-mediated*[tiab] OR computer-generated*[tiab] OR computerized*[tiab] OR computerised*[tiab] OR electronic-based*[tiab] OR electronic-assist*[tiab] OR electronic-mediated*[tiab] OR electronic communicat*[tiab] OR digital device*[tiab] OR digital treat*[tiab] OR digital therap*[tiab] OR digital technolog*[tiab] OR ehealth[tiab] OR e-health[tiab] OR e-treat*[tiab] OR e-therap*[tiab] OR mhealth[tiab] OR m-health[tiab] OR mobile health* [tiab] OR mobile deliver* [tiab] OR mobile-based [tiab] OR internet-based*[tiab] OR internet treat*[tiab] OR internet intervention*[tiab] OR internet counsel*[tiab] OR internet-deliver* [tiab] OR distance counsel*[tiab] OR web-based*[tiab] OR cybercounsel*[tiab] OR cyber-counsel*[tiab] OR online treat*[tiab] OR online therap*[tiab] OR online intervention*[tiab] OR online prevention*[tiab] OR online counsel*[tiab] OR online deliver* [tiab] OR text-messag*[tiab] OR textmessag*[tiab] OR text messag*[tiab] OR SMS[tiab] OR texting*[tiab] OR short message service*[tiab] OR mobile phone*[tiab] OR smartphone*[tiab] OR cell-phone*[tiab] OR cellphone*[tiab] OR blended*[tiab] OR mobile app*[tiab] OR software app*[tiab] OR electronic app*[tiab] OR mobile device*[tiab] OR handheld device*[tiab] OR hand held device*[tiab] OR electronic device*[tiab] OR handheld computer*[tiab] OR hand held computer[tiab] OR iPad*[tiab] OR iPhone*[tiab] OR wearable*[tiab] OR e-counsel*[tiab] OR ecounsel*[tiab] OR palmtop*[tiab] OR digital assist*[tiab] OR telephone*[tiab] OR WhatsApp[tiab] OR “Smartphone”[mesh terms] OR “Telemedicine”[Mesh terms] OR “Text Messaging”[mesh terms] OR “Mobile Applications”[mesh terms]

**Intervention**

“Psychotherapy”[mesh terms] OR psychotherap*[tiab] OR intervention*[tiab] OR treat*[tiab] OR therap*[tiab] OR cognitive behavi*[tiab] OR self-help[tiab] OR counsel*[tiab] OR support[tiab] OR guidance[tiab] OR self-help[tiab] OR ICBT[tiab] OR CBT[tiab] OR CCBT[tiab]

- [AND in builder](https://www.ncbi.nlm.nih.gov/pubmed/advanced)
- [OR in builder](https://www.ncbi.nlm.nih.gov/pubmed/advanced)
- [NOT in builder](https://www.ncbi.nlm.nih.gov/pubmed/advanced)
- [Delete from history](https://www.ncbi.nlm.nih.gov/pubmed/advanced)
- [Show search results](https://www.ncbi.nlm.nih.gov/pubmed/advanced)
- [Show search details](https://www.ncbi.nlm.nih.gov/pubmed/advanced)
- [AND in builder](https://www.ncbi.nlm.nih.gov/pubmed/advanced)
- [OR in builder](https://www.ncbi.nlm.nih.gov/pubmed/advanced)
- [NOT in builder](https://www.ncbi.nlm.nih.gov/pubmed/advanced)
- [Delete from history](https://www.ncbi.nlm.nih.gov/pubmed/advanced)
- [Show search results](https://www.ncbi.nlm.nih.gov/pubmed/advanced)
- [Show search details](https://www.ncbi.nlm.nih.gov/pubmed/advanced)
- [Save in My NCBI](https://www.ncbi.nlm.nih.gov/pubmed/advanced)
- [AND in builder](https://www.ncbi.nlm.nih.gov/pubmed/advanced)
- [OR in builder](https://www.ncbi.nlm.nih.gov/pubmed/advanced)
- [NOT in builder](https://www.ncbi.nlm.nih.gov/pubmed/advanced)
- [Show search results](https://www.ncbi.nlm.nih.gov/pubmed/advanced)
- [Save as a My NCBI Collection](https://www.ncbi.nlm.nih.gov/pubmed/advanced)

**Embase**

**Depression/Anxiety**'depression'/exp OR depress*:ab,ti,kw OR 'mood disorder'/exp OR 'anxiety'/exp OR 'anxiety disorder'/de OR 'anxiety neurosis'/exp OR 'generalized anxiety disorder'/exp OR 'mixed anxiety and depression'/exp OR 'obsessive compulsive disorder'/exp OR 'phobia'/exp OR 'separation anxiety'/exp OR dysthymi*:ab,ti,kw OR anxi*:ab,ti,kw OR ‘social anxi*’:ab,ti,kw OR ‘separation anxi*’:ab,ti,kw OR phobi*:ab,ti,kw OR ‘generalised anxiety disorder*’:ab,ti,kw OR ‘generalized anxiety disorder*’:ab,ti,kw OR OCD:ab,ti,kw OR ‘obsessive compulsive disorder*’:ab,ti,kw OR hoarding:ab,ti,kw

**Population**'juvenile'/de OR 'adolescent'/exp OR 'child'/exp OR 'young adult'/exp OR child:ab,ti,kw OR children:ab,ti,kw OR childhood:ab,ti,kw OR teen:ab,ti,kw OR teens:ab,ti,kw OR teenager*:ab,ti,kw OR adolescen*:ab,ti,kw OR ‘young per*’:ab,ti,kw OR youth*:ab,ti,kw OR boy:ab,ti,kw OR boys:ab,ti,kw OR girl:ab,ti,kw OR girls:ab,ti,kw OR ‘young adult*’:ab,ti,kw OR juvenile*:ab,ti,kw OR ‘young people’:ab,ti,kw OR youngsters:ab,ti,kw OR student*:ab,ti,kw OR college:ab,ti,kw OR schoolchild*:ab,ti,kw OR preadolescen*:ab,ti,kw OR ‘junior high*’:ab,ti,kw OR highschool*:ab,ti,kw OR ‘senior high’:ab,ti,kw OR minors:ab,ti,kw OR boyhood:ab,ti,kw OR girlhood:ab,ti,kw

**E-Health**

'mobile phone'/exp OR 'mobile application'/exp OR 'telehealth'/exp OR 'text messaging'/exp OR ‘web-assist*’:ab,ti,kw OR ‘web-deliver*’:ab,ti,kw OR ‘web-mediated*’:ab,ti,kw OR ‘web-based’:ab,ti,kw OR ‘mobile app’:ab,ti,kw OR telehealth:ab,ti,kw OR ‘tele-health’:ab,ti,kw OR telepsychiatry:ab,ti,kw OR ‘tele-psychiatry’:ab,ti,kw OR ‘tele-therap*’:ab,ti,kw OR teletherap*:ab,ti,kw OR ‘tele-medicine’:ab,ti,kw OR telemedicine:ab,ti,kw OR ‘telecare’:ab,ti,kw OR ‘tele-care’:ab,ti,kw OR ‘computer-based*’:ab,ti,kw OR ‘computer-deliver*’:ab,ti,kw OR ‘computer-assist*’:ab,ti,kw OR ‘computer-mediated*’:ab,ti,kw OR ‘computer-generated*’:ab,ti,kw OR computerized*:ab,ti,kw OR computerised*:ab,ti,kw OR ‘electronic-based*’:ab,ti,kw OR ‘electronic-assist*’:ab,ti,kw OR ‘electronic-mediated*’:ab,ti,kw OR ‘electronic communicat*’:ab,ti,kw OR ‘digital device*’:ab,ti,kw OR ‘digital treat*’:ab,ti,kw OR ‘digital therap*’:ab,ti,kw OR ‘digital technolog*’:ab,ti,kw OR ehealth:ab,ti,kw OR ‘e-health’:ab,ti,kw OR ‘e-treat*’:ab,ti,kw OR ‘e-therap*’:ab,ti,kw OR mhealth:ab,ti,kw OR ‘m-health’:ab,ti,kw OR ‘mobile health*’:ab,ti,kw OR ‘mobile deliver*’:ab,ti,kw OR ‘mobile-based’:ab,ti,kw OR ‘internet-based*’:ab,ti,kw OR ‘internet treat*’:ab,ti,kw OR ‘internet intervention*’:ab,ti,kw OR ‘internet counsel*’:ab,ti,kw OR ‘internet-deliver*’:ab,ti,kw OR ‘distance counsel*’:ab,ti,kw OR ‘web-based*’:ab,ti,kw OR cybercounsel*:ab,ti,kw OR ‘cyber-counsel*’:ab,ti,kw OR ‘online treat*’:ab,ti,kw OR ‘online therap*’:ab,ti,kw OR ‘online intervention*’:ab,ti,kw OR ‘online prevention*’:ab,ti,kw OR ‘online counsel*’:ab,ti,kw OR ‘online deliver*’:ab,ti,kw OR ‘text-messag*’:ab,ti,kw OR textmessag*:ab,ti,kw OR SMS:ab,ti,kw OR texting*:ab,ti,kw OR ‘short message service*’:ab,ti,kw OR ‘mobile phone*’:ab,ti,kw OR smartphone*:ab,ti,kw OR ‘cell-phone*’:ab,ti,kw OR cellphone*:ab,ti,kw OR blended*:ab,ti,kw OR ‘mobile app*’:ab,ti,kw OR ‘software app*’:ab,ti,kw OR ‘electronic app*’:ab,ti,kw OR ‘mobile device*’:ab,ti,kw OR ‘handheld device*’:ab,ti,kw OR ‘hand held device*’:ab,ti,kw OR ‘electronic device*’:ab,ti,kw OR ‘handheld computer*’:ab,ti,kw OR ‘hand held computer’:ab,ti,kw OR iPad*:ab,ti,kw OR iPhone*:ab,ti,kw OR wearable*:ab,ti,kw OR ‘e-counsel*’:ab,ti,kw OR ecounsel*:ab,ti,kw OR palmtop*:ab,ti,kw OR ‘digital assist*’:ab,ti,kw OR telephone*:ab,ti,kw OR WhatsApp:ab,ti,kw

**Intervention**

'psychotherapy'/exp OR psychotherap*:ab,ti,kw OR intervention*:ab,ti,kw OR treat*:ab,ti,kw OR therap*:ab,ti,kw OR ‘cognitive behavi*’:ab,ti,kw OR ‘self-help’:ab,ti,kw OR counsel*:ab,ti,kw OR support:ab,ti,kw OR guidance:ab,ti,kw OR ‘self-help’:ab,ti,kw OR ICBT:ab,ti,kw OR CBT:ab,ti,kw OR CCBT:ab,ti,kw

**PsycINFO (EBSCO)**

**Depression/Anxiety**DE "Major Depression" OR DE "Endogenous Depression" OR DE "Recurrent Depression" OR DE "Treatment Resistant Depression" OR DE "Atypical Depression" OR DE "Depression (Emotion)" OR DE "Seasonal Affective Disorder" OR DE "Generalized Anxiety Disorder" OR DE "Separation Anxiety Disorder" OR DE "Obsessive Compulsive Disorder" OR DE "Hoarding Behavior" OR DE "Hoarding Disorder" OR DE "Social Anxiety" OR DE "Phobias" OR DE "Anxiety Disorders" OR DE "Social Phobia" OR DE "Anxiety" OR DE “School Phobia” OR TI(depress* OR dysthymi* OR anxi* OR “social anxi*” OR “separation anxi*” OR phobi* OR “generalised anxiety disorder*” OR “generalized anxiety disorder*” OR OCD OR “obsessive compulsive disorder*” OR hoarding) OR AB(depress* OR dysthymi* OR anxi* OR “social anxi*” OR “separation anxi*” OR phobi* OR “generalised anxiety disorder*” OR “generalized anxiety disorder*” OR OCD OR “obsessive compulsive disorder*” OR hoarding) OR KW(depress* OR dysthymi* OR anxi* OR phobi* OR OCD OR hoarding)

**Population**TI(child OR children OR childhood OR teen OR teens OR teenager* OR adolescen* OR “young per*” OR youth* OR boy OR boys OR girl OR girls OR “young adult*” OR juvenile* OR “young people” OR youngsters OR student* OR college OR schoolchild* OR preadolescen* OR “junior high*” OR highschool* OR “senior high” OR minors OR boyhood OR girlhood) OR AB(child OR children OR childhood OR teen OR teens OR teenager* OR adolescen* OR “young per*” OR youth* OR boy OR boys OR girl OR girls OR “young adult*” OR juvenile* OR “young people” OR youngsters OR student* OR college OR schoolchild* OR preadolescen* OR “junior high*” OR highschool* OR “senior high” OR minors OR boyhood OR girlhood) OR KW(child OR children OR childhood OR teen OR teens OR teenager* OR adolescen* OR youth* OR boy OR boys OR girl OR girls OR juvenile* OR youngsters OR student* OR college OR schoolchild* OR preadolescen* OR highschool* OR minors OR boyhood OR girlhood)

OR Limiters - Age Groups: Childhood (birth-12 yrs), School Age (6-12 yrs), Adolescence (13-17 yrs), Young Adulthood (18-29 yrs)

**E-Health**

DE "Cellular Phones" OR DE "Text Messaging" OR DE "Mobile Devices" OR DE "Telemedicine" OR DE "Computer Assisted Therapy" OR TI(“web-assist*” OR “web-deliver*” OR “web-mediated*” OR “web-based” OR “mobile app” OR telehealth OR “tele-health” OR telepsychiatry OR “tele-psychiatry” OR “tele-therap*” OR teletherap* OR “tele-medicine” OR telemedicine OR “telecare” OR “tele-care” OR “computer-based*” OR “computer-deliver*” OR “computer-assist*” OR “computer-mediated*” OR “computer-generated*” OR computerized* OR computerised* OR “electronic-based*” OR “electronic-assist*” OR “electronic-mediated*” OR “electronic communicat*” OR “digital device*” OR “digital treat*” OR “digital therap*” OR “digital technolog*” OR ehealth OR “e-health” OR “e-treat*” OR “e-therap*” OR mhealth OR “m-health” OR “mobile health*” OR “mobile deliver*” OR “mobile-based” OR “internet-based*” OR “internet treat*” OR “internet intervention*” OR “internet counsel*” OR “internet-deliver*” OR “distance counsel*” OR “web-based*” OR cybercounsel* OR “cyber-counsel*” OR “online treat*” OR “online therap*” OR “online intervention*” OR “online prevention*” OR “online counsel*” OR “online deliver*” OR “text-messag*” OR textmessag* OR SMS OR texting* OR “short message service*” OR “mobile phone*” OR smartphone* OR “cell-phone*” OR cellphone* OR blended* OR “mobile app*” OR “software app*” OR “electronic app*” OR “mobile device*” OR “handheld device*” OR “hand held device*” OR “electronic device*” OR “handheld computer*” OR “hand held computer” OR iPad* OR iPhone* OR wearable* OR “e-counsel*” OR ecounsel* OR palmtop* OR “digital assist*” OR telephone* OR WhatsApp) OR AB(“web-assist*” OR “web-deliver*” OR “web-mediated*” OR “web-based” OR “mobile app” OR telehealth OR “tele-health” OR telepsychiatry OR “tele-psychiatry” OR “tele-therap*” OR teletherap* OR “tele-medicine” OR telemedicine OR “telecare” OR “tele-care” OR “computer-based*” OR “computer-deliver*” OR “computer-assist*” OR “computer-mediated*” OR “computer-generated*” OR computerized* OR computerised* OR “electronic-based*” OR “electronic-assist*” OR “electronic-mediated*” OR “electronic communicat*” OR “digital device*” OR “digital treat*” OR “digital therap*” OR “digital technolog*” OR ehealth OR “e-health” OR “e-treat*” OR “e-therap*” OR mhealth OR “m-health” OR “mobile health*” OR “mobile deliver*” OR “mobile-based” OR “internet-based*” OR “internet treat*” OR “internet intervention*” OR “internet counsel*” OR “internet-deliver*” OR “distance counsel*” OR “web-based*” OR cybercounsel* OR “cyber-counsel*” OR “online treat*” OR “online therap*” OR “online intervention*” OR “online prevention*” OR “online counsel*” OR “online deliver*” OR “text-messag*” OR textmessag* OR SMS OR texting* OR “short message service*” OR “mobile phone*” OR smartphone* OR “cell-phone*” OR cellphone* OR blended* OR “mobile app*” OR “software app*” OR “electronic app*” OR “mobile device*” OR “handheld device*” OR “hand held device*” OR “electronic device*” OR “handheld computer*” OR “hand held computer” OR iPad* OR iPhone* OR wearable* OR “e-counsel*” OR ecounsel* OR palmtop* OR “digital assist*” OR telephone* OR WhatsApp) OR KW(telehealth OR telepsychiatry OR teletherap* OR telemedicine OR telecare OR computerized* OR computerised* OR ehealth OR mhealth OR texting* OR smartphone* OR cellphone* OR blended* OR iPad* OR iPhone* OR wearable* OR ecounsel* OR palmtop* OR telephone* OR WhatsApp)

**Intervention**

DE "Cognitive Behavior Therapy" OR DE "Psychotherapy" OR DE "Behavior Therapy" OR DE "Cognitive Therapy" OR TI(psychotherap* OR intervention* OR treat* OR therap* OR “cognitive behavi*” OR “self-help” OR counsel* OR support OR guidance OR “self-help” OR ICBT OR CBT OR CCBT) OR AB(psychotherap* OR intervention* OR treat* OR therap* OR “cognitive behavi*” OR “self-help” OR counsel* OR support OR guidance OR “self-help” OR ICBT OR CBT OR CCBT) OR KW(psychotherap* OR intervention* OR treat* OR therap* OR counsel* OR support OR guidance OR ICBT OR CBT OR CCBT)

**Cinahl (EBSCO)**
**Depression/Anxiety**(MH "Affective Disorders+") OR (MH "Anxiety") OR (MM "Separation Anxiety") OR (MM "Depression") OR (MH "Anxiety Disorders+") OR (MH "Obsessive-Compulsive Disorder+") OR (MH "Phobic Disorders+") OR TI(depress* OR dysthymi* OR anxi* OR “social anxi*” OR “separation anxi*” OR phobi* OR “generalised anxiety disorder*” OR “generalized anxiety disorder*” OR OCD OR “obsessive compulsive disorder*” OR hoarding) OR AB(depress* OR dysthymi* OR anxi* OR “social anxi*” OR “separation anxi*” OR phobi* OR “generalised anxiety disorder*” OR “generalized anxiety disorder*” OR OCD OR “obsessive compulsive disorder*” OR hoarding) OR SU(depress* OR dysthymi* OR anxi* OR phobi* OR OCD OR hoarding)

**Population**TI(child OR children OR childhood OR teen OR teens OR teenager* OR adolescen* OR “young per*” OR youth* OR boy OR boys OR girl OR girls OR “young adult*” OR juvenile* OR “young people” OR youngsters OR student* OR college OR schoolchild* OR preadolescen* OR “junior high*” OR highschool* OR “senior high” OR minors OR boyhood OR girlhood) OR AB(child OR children OR childhood OR teen OR teens OR teenager* OR adolescen* OR “young per*” OR youth* OR boy OR boys OR girl OR girls OR “young adult*” OR juvenile* OR “young people” OR youngsters OR student* OR college OR schoolchild* OR preadolescen* OR “junior high*” OR highschool* OR “senior high” OR minors OR boyhood OR girlhood) OR SU(child OR children OR childhood OR teen OR teens OR teenager* OR adolescen* OR youth* OR boy OR boys OR girl OR girls OR juvenile* OR youngsters OR student* OR college OR schoolchild* OR preadolescen* OR highschool* OR minors OR boyhood OR girlhood)

OR Limiters - Age Groups: Childhood (birth-12 yrs), School Age (6-12 yrs), Adolescence (13-17 yrs), Young Adulthood (18-29 yrs)

**E-Health**

(MH "Smartphone+") OR (MH "Mobile Applications") OR (MH "Cellular Phone+") OR (MH "Telehealth+") OR (MH "Therapy, Computer Assisted") OR TI(“web-assist*” OR “web-deliver*” OR “web-mediated*” OR “web-based” OR “mobile app” OR telehealth OR “tele-health” OR telepsychiatry OR “tele-psychiatry” OR “tele-therap*” OR teletherap* OR “tele-medicine” OR telemedicine OR “telecare” OR “tele-care” OR “computer-based*” OR “computer-deliver*” OR “computer-assist*” OR “computer-mediated*” OR “computer-generated*” OR computerized* OR computerised* OR “electronic-based*” OR “electronic-assist*” OR “electronic-mediated*” OR “electronic communicat*” OR “digital device*” OR “digital treat*” OR “digital therap*” OR “digital technolog*” OR ehealth OR “e-health” OR “e-treat*” OR “e-therap*” OR mhealth OR “m-health” OR “mobile health*” OR “mobile deliver*” OR “mobile-based” OR “internet-based*” OR “internet treat*” OR “internet intervention*” OR “internet counsel*” OR “internet-deliver*” OR “distance counsel*” OR “web-based*” OR cybercounsel* OR “cyber-counsel*” OR “online treat*” OR “online therap*” OR “online intervention*” OR “online prevention*” OR “online counsel*” OR “online deliver*” OR “text-messag*” OR textmessag* OR SMS OR texting* OR “short message service*” OR “mobile phone*” OR smartphone* OR “cell-phone*” OR cellphone* OR blended* OR “mobile app*” OR “software app*” OR “electronic app*” OR “mobile device*” OR “handheld device*” OR “hand held device*” OR “electronic device*” OR “handheld computer*” OR “hand held computer” OR iPad* OR iPhone* OR wearable* OR “e-counsel*” OR ecounsel* OR palmtop* OR “digital assist*” OR telephone* OR WhatsApp) OR AB(“web-assist*” OR “web-deliver*” OR “web-mediated*” OR “web-based” OR “mobile app” OR telehealth OR “tele-health” OR telepsychiatry OR “tele-psychiatry” OR “tele-therap*” OR teletherap* OR “tele-medicine” OR telemedicine OR “telecare” OR “tele-care” OR “computer-based*” OR “computer-deliver*” OR “computer-assist*” OR “computer-mediated*” OR “computer-generated*” OR computerized* OR computerised* OR “electronic-based*” OR “electronic-assist*” OR “electronic-mediated*” OR “electronic communicat*” OR “digital device*” OR “digital treat*” OR “digital therap*” OR “digital technolog*” OR ehealth OR “e-health” OR “e-treat*” OR “e-therap*” OR mhealth OR “m-health” OR “mobile health*” OR “mobile deliver*” OR “mobile-based” OR “internet-based*” OR “internet treat*” OR “internet intervention*” OR “internet counsel*” OR “internet-deliver*” OR “distance counsel*” OR “web-based*” OR cybercounsel* OR “cyber-counsel*” OR “online treat*” OR “online therap*” OR “online intervention*” OR “online prevention*” OR “online counsel*” OR “online deliver*” OR “text-messag*” OR textmessag* OR SMS OR texting* OR “short message service*” OR “mobile phone*” OR smartphone* OR “cell-phone*” OR cellphone* OR blended* OR “mobile app*” OR “software app*” OR “electronic app*” OR “mobile device*” OR “handheld device*” OR “hand held device*” OR “electronic device*” OR “handheld computer*” OR “hand held computer” OR iPad* OR iPhone* OR wearable* OR “e-counsel*” OR ecounsel* OR palmtop* OR “digital assist*” OR telephone* OR WhatsApp) OR SU(telehealth OR telepsychiatry OR teletherap* OR telemedicine OR telecare OR computerized* OR computerised* OR ehealth OR mhealth OR texting* OR smartphone* OR cellphone* OR blended* OR iPad* OR iPhone* OR wearable* OR ecounsel* OR palmtop* OR telephone* OR WhatsApp)

**Intervention**

(MH "Psychotherapy+") OR TI(psychotherap* OR intervention* OR treat* OR therap* OR “cognitive behavi*” OR “self-help” OR counsel* OR support OR guidance OR “self-help” OR ICBT OR CBT OR CCBT) OR AB(psychotherap* OR intervention* OR treat* OR therap* OR “cognitive behavi*” OR “self-help” OR counsel* OR support OR guidance OR “self-help” OR ICBT OR CBT OR CCBT) OR SU(psychotherap* OR intervention* OR treat* OR therap* OR counsel* OR support OR guidance OR ICBT OR CBT OR CCBT)

**Web of Science – Clarivate**

**Depression/Anxiety**TS=(“depress*” OR “dysthymi*” OR “anxi*” OR “social anxi*” OR “separation anxi*” OR “phobi*” OR “generalised anxiety disorder*” OR “generalized anxiety disorder*” OR “OCD” OR “obsessive compulsive disorder*” OR “hoarding”)

**Population**

TS=(“child” OR “children” OR “childhood” OR “teen” OR “teens” OR “teenager*” OR “adolescen*” OR “young per*” OR “youth*” OR “boy” OR “boys” OR “girl” OR “girls” OR “young adult*” OR “juvenile*” OR “young people” OR “youngsters” OR “student*” OR “college” OR “schoolchild*” OR “preadolescen*” OR “junior high*” OR highschool* OR “senior high” OR “minors” OR “boyhood” OR “girlhood”)

**E-Health**

TS=(“web-assist*” OR “web-deliver*” OR “web-mediated*” OR “web-based” OR “mobile app” OR “telehealth” OR “tele-health” OR “telepsychiatry” OR “tele-psychiatry” OR “tele-therap*” OR “teletherap*” OR “tele-medicine” OR “telemedicine” OR “telecare” OR “tele-care” OR “computer-based*” OR “computer-deliver*” OR “computer-assist*” OR “computer-mediated*” OR “computer-generated*” OR “computerized*” OR “computerised*” OR “electronic-based*” OR “electronic-assist*” OR “electronic-mediated*” OR “electronic communicat*” OR “digital device*” OR “digital treat*” OR “digital therap*” OR “digital technolog*” OR “ehealth” OR “e-health” OR “e-treat*” OR “e-therap*” OR “mhealth” OR “m-health” OR “mobile health*” OR “mobile deliver*” OR “mobile-based” OR “internet-based*” OR “internet treat*” OR “internet intervention*” OR “internet counsel*” OR “internet-deliver*” OR “distance counsel*” OR “web-based*” OR “cybercounsel*” OR “cyber-counsel*” OR “online treat*” OR “online therap*” OR “online intervention*” OR “online prevention*” OR “online counsel*” OR “online deliver*” OR “text-messag*” OR “textmessag*” OR “SMS” OR “texting*” OR “short message service*” OR “mobile phone*” OR “smartphone*” OR “cell-phone*” OR “cellphone*” OR “blended*” OR “mobile app*” OR “software app*” OR “electronic app*” OR “mobile device*” OR “handheld device*” OR “hand held device*” OR “electronic device*” OR “handheld computer*” OR “hand held computer” OR “iPad*” OR “iPhone*” OR “wearable*” OR “e-counsel*” OR “ecounsel*” OR “palmtop*” OR “digital assist*” OR “telephone*” OR “WhatsApp”)

**Intervention**TS=(“psychotherap*” OR “intervention*” OR “treat*” OR “therap*” OR “cognitive behavi*” OR “self-help” OR “counsel*” OR “support” OR “guidance” OR “self-help” OR “ICBT” OR “CBT” OR “CCBT”)

Top of Form

Bottom of Form

**Cochrane (Wiley)**

**Depression/Anxiety**(“depress*” OR “dysthymi*” OR “anxi*” OR “social anxi*” OR “separation anxi*” OR “phobi*” OR “generalised anxiety disorder*” OR “generalized anxiety disorder*” OR “OCD” OR “obsessive compulsive disorder*” OR “hoarding”):ab,ti,kw

**Population**

(“child” OR “children” OR “childhood” OR “teen” OR “teens” OR “teenager*” OR “adolescen*” OR “young per*” OR “youth*” OR “boy” OR “boys” OR “girl” OR “girls” OR “young adult*” OR “juvenile*” OR “young people” OR “youngsters” OR “student*” OR “college” OR “schoolchild*” OR “preadolescen*” OR “junior high*” OR highschool* OR “senior high” OR “minors” OR “boyhood” OR “girlhood”):ab,ti,kw

**E-Health**

(“web-assist*” OR “web-deliver*” OR “web-mediated*” OR “web-based” OR “mobile app” OR “telehealth” OR “tele-health” OR “telepsychiatry” OR “tele-psychiatry” OR “tele-therap*” OR “teletherap*” OR “tele-medicine” OR “telemedicine” OR “telecare” OR “tele-care” OR “computer-based*” OR “computer-deliver*” OR “computer-assist*” OR “computer-mediated*” OR “computer-generated*” OR “computerized*” OR “computerised*” OR “electronic-based*” OR “electronic-assist*” OR “electronic-mediated*” OR “electronic communicat*” OR “digital device*” OR “digital treat*” OR “digital therap*” OR “digital technolog*” OR “ehealth” OR “e-health” OR “e-treat*” OR “e-therap*” OR “mhealth” OR “m-health” OR “mobile health*” OR “mobile deliver*” OR “mobile-based” OR “internet-based*” OR “internet treat*” OR “internet intervention*” OR “internet counsel*” OR “internet-deliver*” OR “distance counsel*” OR “web-based*” OR “cybercounsel*” OR “cyber-counsel*” OR “online treat*” OR “online therap*” OR “online intervention*” OR “online prevention*” OR “online counsel*” OR “online deliver*” OR “text-messag*” OR “textmessag*” OR “SMS” OR “texting*” OR “short message service*” OR “mobile phone*” OR “smartphone*” OR “cell-phone*” OR “cellphone*” OR “blended*” OR “mobile app*” OR “software app*” OR “electronic app*” OR “mobile device*” OR “handheld device*” OR “hand held device*” OR “electronic device*” OR “handheld computer*” OR “hand held computer” OR “iPad*” OR “iPhone*” OR “wearable*” OR “e-counsel*” OR “ecounsel*” OR “palmtop*” OR “digital assist*” OR “telephone*” OR “WhatsApp”):ab,ti,kw

**Intervention**(“psychotherap*” OR “intervention*” OR “treat*” OR “therap*” OR “cognitive behavi*” OR “self-help” OR “counsel*” OR “support” OR “guidance” OR “self-help” OR “ICBT” OR “CBT” OR “CCBT”):ab,ti,kw
