# Supplementary material for: Internet and Computer-Based Cognitive Behavioral Therapy for Anxiety and Depression in Adolescents and Young Adults: Systematic Review and Meta-Analysis
Source: J Med Internet Res. 2020 Sep 25;22(9):e17831. doi: 10.2196/17831 (PMC7547394; doi:10.2196/17831)
Supplement: Multimedia Appendix 2 [file jmir_v22i9e17831_app2.docx]

**Multimedia Appendix**

*Selected characteristics of the included studies examining the effects of cCBT for depression and anxiety in adolescents and young adults.*

| Study | Focus | Recruit. | *M* age (*SD*) | Age range | % Fem. | Diag./elev. | Intervention | Control | N_i_ rand. (analyzed) | N_c_ rand. (analyzed) | Guided | Primary outcome | Country |
| --- | --- | --- | --- | --- | --- | --- | --- | --- | --- | --- | --- | --- | --- |
| Botella 2010 | Anx. | Univ. | 24.40 (5.78) | 18-48 | 79.2 | Diag. | iCBT  *Talk to me* | 1: FTF CBT 2: Waiting list | 62 (62) | 1: 36 (36) 2: 29 (29) | No | D: BDI-13(SR) A: BFNE(SR) | Spain |
| Bowler 2012 | Anx. | Univ. | 21.90 (4.42) | 18-48 | 64.3 | Elev. | iCBT | Waiting list | 24 (21) | 22 (21) | No | D: BDI-II(SR) A: FNE(SR) | UK |
| Clarke  2009 | Depr. | Clin. | 22.6 (2.6) | 18-24 | 79 | Diag. | iCBT | Information control | 56 (56) | 53 (53) | No | D: PHQ-8(SR) A: N.a. | USA |
| Day 2013 | Anx./ Depr./ Stress | Univ. | 23.55 (4.98) | 18-45 | 89.3 | Elev. | iCBT | Waiting list | 33 (33) | 33 (33) | Yes | D: DASS-21(SR) A: DASS-21(SR) | Canada |
| Ellis  2011 | Anx./ Depr. | Univ. | 19.67 (1.66) | 18-25 | 77.0 | Elev. | iCBT  *MoodGym* | No treatment | 13 (13) | 13 (13) | Yes | D: DASS-21(SR) A: DASS-21(SR) | Australia |
| Fitzpatrick 2017 | Anx./ Depr. | Univ. | 22.2 (2.33) | 18-28 | 81.0 | Elev. | cCBT *Woebot* | Information control | 34 (34) | 36 (36) | No | D: PHQ-9(SR) A: GAD-7(SR) | USA |
| Fleming 2012 | Depr. | Educ. Progr. | 14.9 (0.79) | 13-16 | 44.0 | Elev. | cCBT *SPARX* | Waiting list | 20 (19) | 12 (11) | No | D: CDRS-R(OR) A: SCAS(SR) | New  Zealand |
| Ip  2016 | Depr. | Sec. school | 14.63 (0.81) | 13-17 | 68.1 | Elev. | iCBT  *Grasp the Opportunity* | Information control | 130 (123) | 127 (127) | No | D: CES-D(SR) A: DASS-21(SR) | China |
| Lenhard 2017 | Anx. | Comm. | 14.60 (1.71) | 12-17 | 46 | Diag. | iCBT  *BiP OCD* | Waiting list | 33 (33) | 34 (34) | Yes | D: CDI-S(SR) A: CY-BOCS(OR) | Sweden |
| McCall  2018 | Anx. | Univ. | 21.86 (5.50) | 17-46 | 72 | Elev. | iCBT  *Overcome Social Anxiety* | Waiting list | 51 (30) | 50 (35) | No | D: N.a. A: FNE(SR) | Canada |
| Merry  2012 | Depr. | Clin. | 15.56 (1.60) | 12-19 | 65.8 | Elev. | cCBT  *SPARX* | TAU | 94 (94) | 93 (93) | No | D: CDRS-R(OR) A: SCAS-C(SR) | New  Zealand |
| Poppelaars2016 | Depr. | Sec. school | 13.35 (0.71) | 11-16 | 100 | Elev. | cCBT  *SPARX* | 1: FTF CBT 2: Waiting list | 51 (51) | 1: 50 (50) 2: 51 (51) | No | D: RADS-2(SR) A: N.a. | Nether-lands |
| Richards 2016 | Anx. | Univ. | 23.82 (7.05) | 17-58 | 77.4 | Elev. | iCBT  *Calming Anxiety* | Waiting list | 70 (70) | 67 (67) | Yes | D: BDI-2(SR) A: GAD-7(SR) | Ireland |
| Sethi  2010 | Anx./ Depr. | Univ. | 19.47 (1.57) | 18-23 | 72.4 | Elev. | iCBT  *MoodGym* | 1: FTF CBT 2: Waiting list | 9 (9) | 1: 10 (10) 2: 10 (10) | Yes | D: DASS-21(SR) A: DASS-21(SR) | Australia |
| Sethi  2013 | Anx./ Depr. | Univ. + Comm. | 20.19 (1.29) | 18-25 | 67.2 | Elev. | iCBT  *MoodGym* | 1: FTF CBT 2: Waiting list | 23 (23) | 1: 21 (21) 2: 23 (23) | Yes | D: DASS-21(SR) A: DASS-21(SR) | Australia |
| Smith 2015 | Depr. | Sec. school | 13.31 (1.24) | 12-16 | 57.0 | Elev. | cCBT *Stressbusters* | Waiting list | 55 (55) | 57 (55) | No | D: MFQ-C(SR) A: SCARED(SR) | UK |
| Spence 2011 | Anx. | Comm. | 13.98 (1.63) | 12-18 | 59 | Diag. | iCBT  *BRAVE* | 1: FTF CBT 2: Waiting list | 44 (44) | 1: 44 (44) 2: 27 (27) | Yes | D: N.a. A: SCAS-C(SR) | Australia |
| Stallard 2011 | Depr./Anx. | Clin. | Mean: N.R.  Median_i_:12 Median_c_:15 | 11-17 | 33 | Diag. or Elev. | cCBT  *Think, feel, do* | Waiting list | 10 (10) | 10 (10) | Yes | D: AWS(SR) A: SCAS-C(SR) | UK |
| Stjerneklar2019 | Anx. | Comm. | 15.03 (1.30) | 13-17 | 79 | Diag. | iCBT  *ChilledOut Online* | Waiting list | 35 (32) | 35 (31) | Yes | D: S-MFQ(SR) A: SCAS-C(SR) | Denmark |
| Tillfors  2011 | Anx. | Comm. | 16.5 (1.6) | 15-21 | 89 | Diag. | iCBT | Waiting list | 10 (9) | 9 (9) | Yes | D: MADRS (SR) A: SPSQ-C (SR) | Sweden |
| Topooco 2018 | Depr. | Comm. | 17.04 (1.1) | 15-19 | 94.3 | Elev. | iCBT | Attention control | 33 (33) | 37 (37) | Yes | D: BDI-II (SR) A: BAI(SR) | Sweden |
| Van der Zanden 2012 | Depr. | Comm. | 20.9 (2.2) | 16-25 | 84.4 | Elev. | iCBT  *Master Your Mood* | Waiting list | 121 (121) | 123 (123) | Yes | D: CES-D (SR) A: HADS Anx.(SR) | Nether-lands |
| Waite 2019 | Anx. | Clin. | 14.7 (1.42) | 13-18 | 65.0 | Diag. | iCBT *BRAVE for teenagers ONLINE* | Waiting list | 30 (30) | 30 (30) | Yes | D: SMFQ-C(SR) A: SCAS-C(SR) | UK |
| Wuthrich 2012 | Anx. | Comm. | 15.55 (1.11) | 14-17 | 62.8 | Diag. | cCBT *Cool Teens* | Waiting list | 24 (24) | 19 (19) | Yes | D: N.a. A: SCAS-C(SR) | Australia |

*Abbreviations*: A = Anxiety; ADIS = Anxiety Disorders Interview Schedule for DSM-IV – child version; Anx. = anxiety; AWS = Adolescent Well Being Scale; BAI = Beck Anxiety Inventory; BFNE = Brief version of the Fear of Negative Evaluation Scale; BDI-13 = Beck Depression Inventory short form; BDI-II = Beck Depression Inventory-II; cCBT = computer-based Cognitive Behavioral Therapy; CDI-S = Child Depression Inventory short version; CDRS-R = Children’s Depression Rating Scale, Revised; CESD-R = Center for Epidemiologic Studies Depression Scale – Revised; Clin. = clinical; Comm. = community; CY-BOCS = Children’s Yale-Brown Obsessive-Compulsive Scale; D = Depression; DASS-21 = Depression Anxiety and Stress Scale-21; Depr. = depression; Diag./Elev. = diagnosis or elevated symptoms required; Educ. progr. = educational program; Fem. = female; FNE = Fear of Negative Evaluation scale; FTF = face-to-face; GAD-7 = Generalized Anxiety Disorder 7-item scale; HADS = Hospital Anxiety and Depression Scale (Anxiety subscale); iCBT = internet-based Cognitive Behavioral Therapy; MADRS-S = Montgomery-Asberg Depression Rating Scale self-report version; MFQ-C = Mood and Feelings Questionnaire – Child report; N.a. = not applicable; N_c_ rand. = randomized N of control group; N_i_ rand. = randomized N of intervention group; N_mod_ = Number of cCBT modules; N.R. = not reported; OR = observer-rated; PHQ-8 = Patient Health Questionnaire-8; PHQ-9 = Patient Health Questionnaire-9; RADS-2 = Reynolds Adolescent Depression Scale-2; Recruit. = recruitment; SCARED = Screen for Child Anxiety Related Disorders; SCAS = Spence Children’s Anxiety Scale; Sec. schools = secondary schools; S-MFQ = Short version of the Mood and Feelings Questionnaire; SPSQ-C = Social Phobia Screening Questionnaire for Children; SR = self-report; TAU = Treatment As Usual; UK = United Kingdom; Univ. = university; USA = United States of America.
