# Supplementary material for: Internet and Computer-Based Cognitive Behavioral Therapy for Anxiety and Depression in Adolescents and Young Adults: Systematic Review and Meta-Analysis
Source: J Med Internet Res. 2020 Sep 25;22(9):e17831. doi: 10.2196/17831 (PMC7547394; doi:10.2196/17831)
Supplement: Multimedia Appendix 3 [file jmir_v22i9e17831_app3.docx]

**Multimedia Appendix**

*Adherence rates of the included studies examining the effects of cCBT for depression and anxiety in adolescents and young adults.*

| Study | Intervention | Number of modules | N_i_ reported (randomized) | Mean % of completed sessions | % of N_i_ that completed  all sessions | Adherence  rating high/low^a^ |  |
| --- | --- | --- | --- | --- | --- | --- | --- |
| Botella 2010 | iCBT  *Talk to me* | N.R. | N.R. (62) | N.R. | N.R. | N.R. |  |
| Bowler 2012 | iCBT | 4 | 21 (24) | 100% | 100% | High |  |
| Clarke 2009 | iCBT | N.A.^b^ | N.A. ^b^ (56) | N.A. ^b^ | N.A. ^b^ | N.A. ^b^ |  |
| Day 2013 | iCBT | 5 | 33 (33) | N.R. | 61% | High |  |
| Ellis 2011 | iCBT  *MoodGym* | 3 | N.R. (13) | N.R. | N.R. | N.R. |  |
| Fitzpatrick 2017 | cCBT *Woebot* | 1-20 | N.A.^c^ (34) | N.A. ^c^ | N.A. ^c^ | N.A. ^c^ |  |
| Fleming 2012 | cCBT *SPARX* | 7 | 20 (20) | N.R. | 75% | High |  |
| Ip 2016 | iCBT  *Grasp the Opportunity* | 10 | 130 (130) | N.R. | 10% | Low |  |
| Lenhard 2017 | iCBT  *BiP OCD* | 12 | 33 (33) | 71% | 27% | Low |  |
| McCall 2018 | iCBT  *Overcome Social Anxiety* | 7 | N.R. (51) | N.R. | N.R. | N.R. |  |
| Merry 2012 | cCBT  *SPARX* | 7 | 94 (94) | N.R. | 60% | High |  |
| Poppelaars 2016 | cCBT  *SPARX* | 7 | 107 ^d^ (51) | 93% ^d^ | 78.8% ^d^ | High ^d^ |  |
| Richards 2016 | iCBT  *Calming Anxiety* | 6 | 70 (70) | N.R. | 18.52% | Low |  |
| Sethi 2010 | iCBT  *MoodGym* | 3 | 9 (9) | 100% | 100% | High |  |
| Sethi 2013 | iCBT  *MoodGym* | 5 | 23 (23) | 100% | 100% | High |  |
| Smith 2015 | cCBT  *Stressbusters* | 8 | 55 (55) | N.R. | 86% | High |  |
| Spence 2011 | iCBT  *BRAVE* | 10 | 44 (44) | 75% | 39% | Low |  |
| Stallard 2011 | cCBT  *Think, feel, do* | 6 | 10 (10) | N.R. | 60% | High |  |
| Stjerneklar 2019 | iCBT  *ChilledOut Online* | 8 | 35 (35) | 63.6% | 28.6% | Low |  |
| Tillfors 2011 | iCBT | 9 | 10 (10) | 32.2% | 0% | Low |  |
| Topooco 2018 | iCBT | 8 | 33 (33) | 81% | 54.5% | High |  |
| Van der Zanden 2012 | iCBT  *Master Your Mood* | 6 | 121 (121) | 53.3% | 20% | Low |  |
| Waite 2019 | iCBT *BRAVE for teenagers ONLINE* | 10 | 58 ^e^ (30) | N.R. | 79.3% ^e^ | High ^e^ |  |
| Wuthrich 2012 | cCBT *Cool Teens* | 8 | 24 (24) | N.R. | 87.5% | High |  |

*Abbreviations*: cCBT = computer-based Cognitive Behavioral Therapy; iCBT = internet-based Cognitive Behavioral Therapy; N.A. = not applicable; N_i_ = N of intervention group; N.R. = not reported.

*Notes:*
^a^: <50% = low adherence; $\geq$50% = high adherence.
^b^: Intervention concerns a website with no specific amount of sessions that are to be completed.

^c^: Intervention concerns conversations in Woebot, an automated conversational agent, with no specific amount of sessions that are to be completed.

^d^: Adherence rates contain two intervention groups, of which only the SPARX only group (n = 51) was included in our meta-analysis.
^e^: Adherence rates contain both intervention group (n = 30) and waiting list group (n = 28).
