# Supplementary figures and images for: Internet and Computer-Based Cognitive Behavioral Therapy for Anxiety and Depression in Adolescents and Young Adults: Systematic Review and Meta-Analysis
Source: J Med Internet Res. 2020 Sep 25;22(9):e17831. doi: 10.2196/17831 (PMC7547394; doi:10.2196/17831)

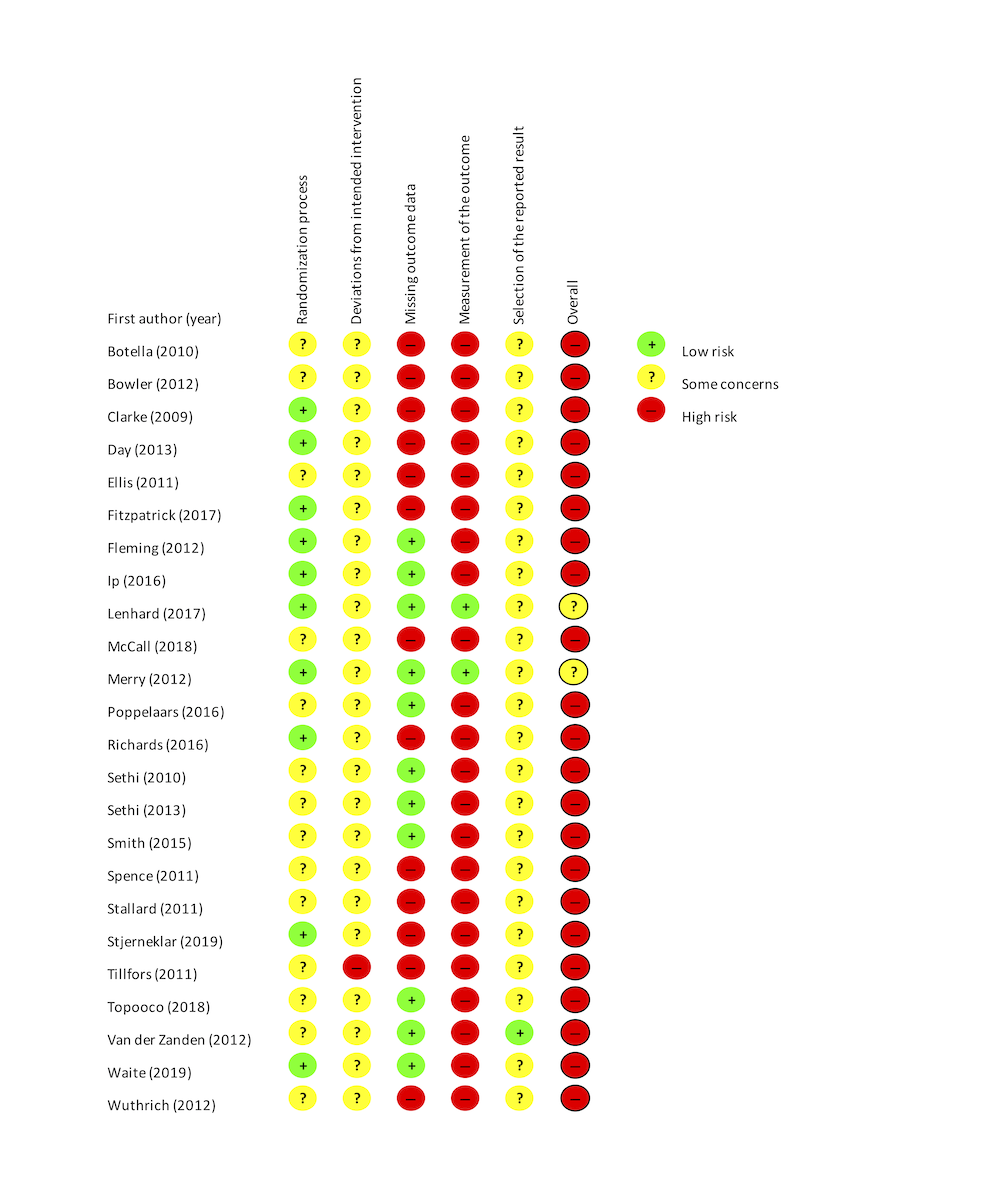

Supplement: Multimedia Appendix 4 [file jmir_v22i9e17831_app4.png]

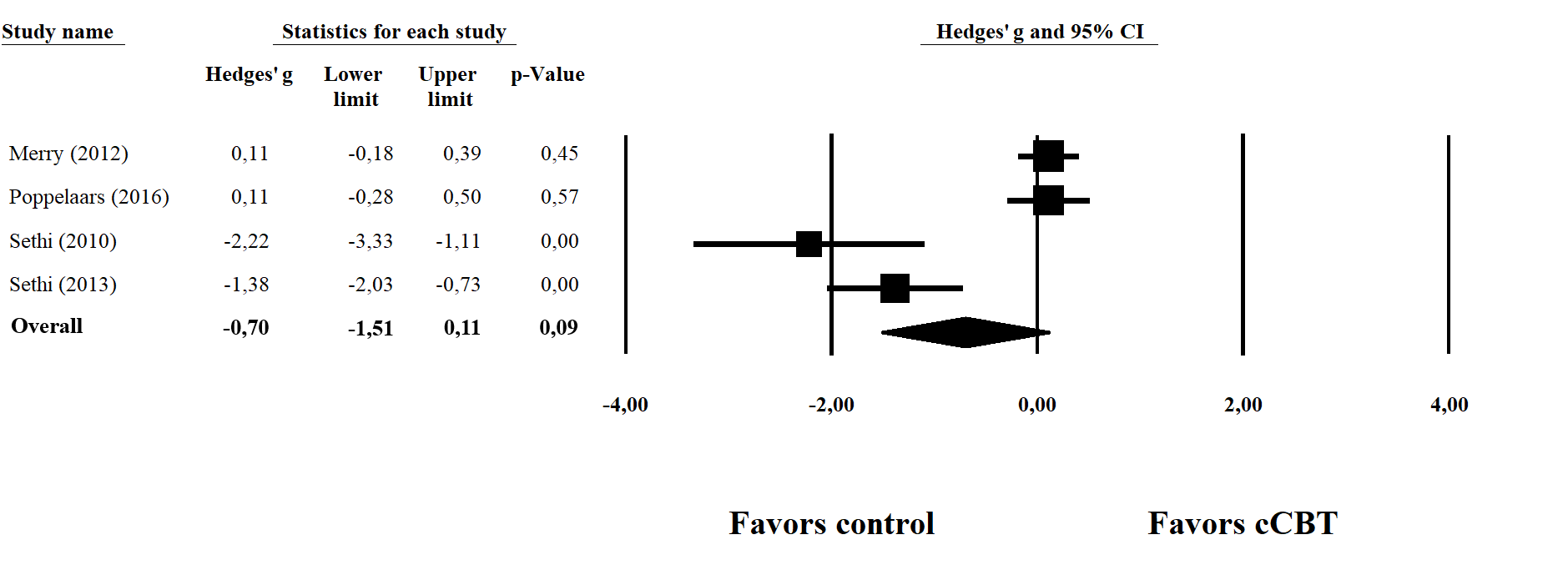

Supplement: Multimedia Appendix 5 [file jmir_v22i9e17831_app5.png]

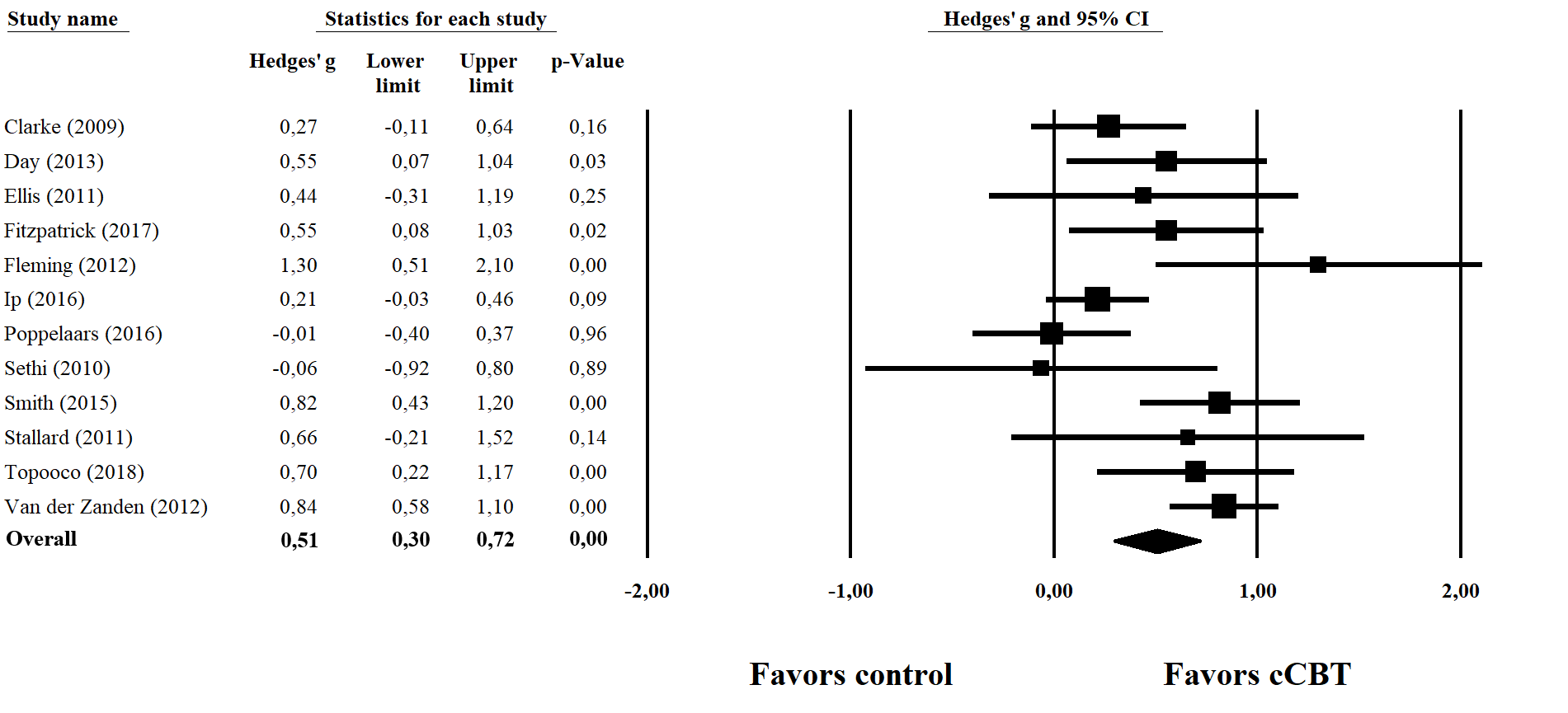

Supplement: Multimedia Appendix 6 [file jmir_v22i9e17831_app6.png]

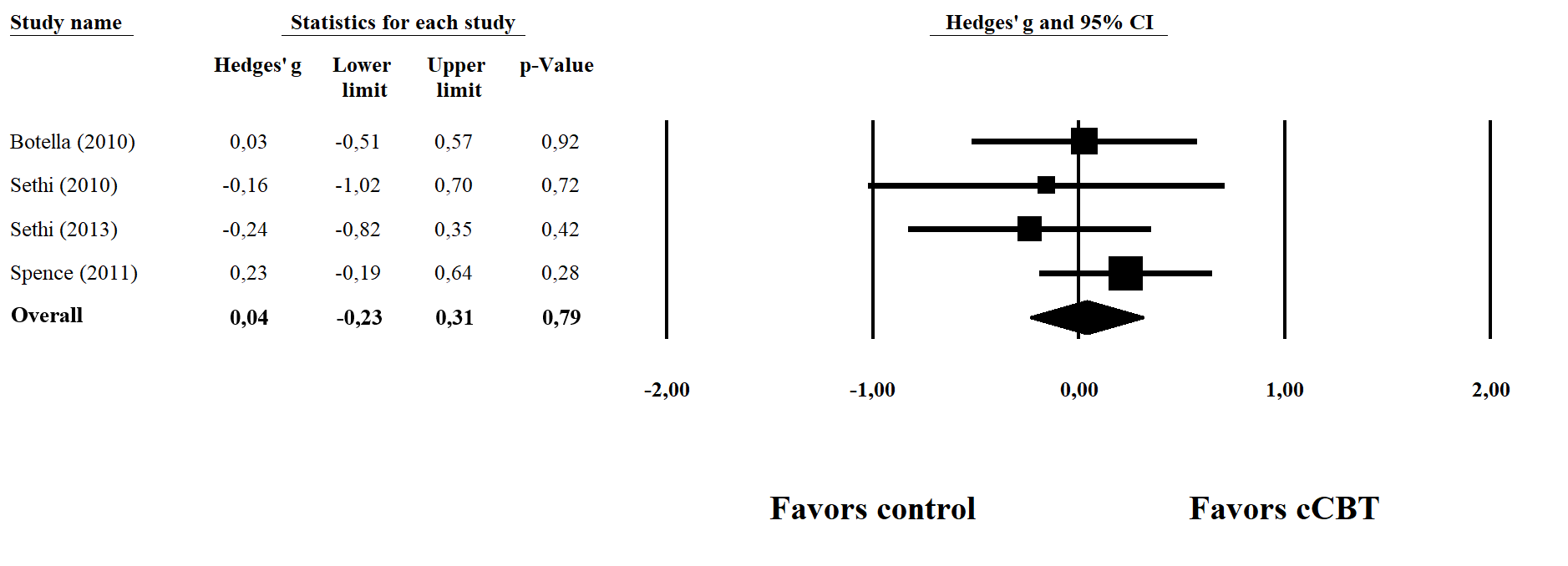

Supplement: Multimedia Appendix 7 [file jmir_v22i9e17831_app7.png]

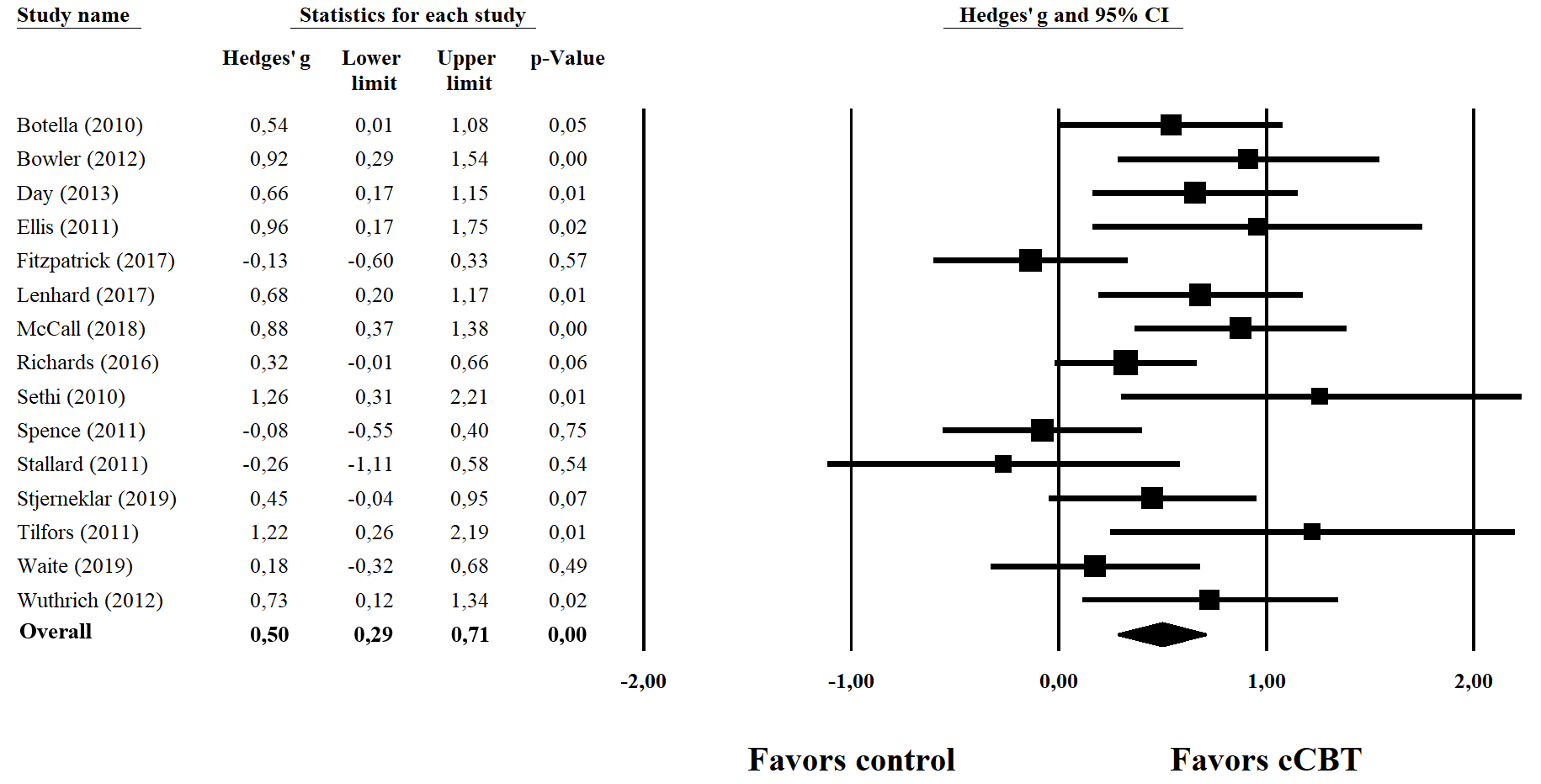

Supplement: Multimedia Appendix 8 [file jmir_v22i9e17831_app8.png]
